# Deficiency of CFB attenuates renal tubulointerstitial damage by inhibiting ceramide synthesis in diabetic kidney disease

**DOI:** 10.1172/jci.insight.156748

**Published:** 2022-12-22

**Authors:** Zi-jun Sun, Dong-yuan Chang, Min Chen, Ming-hui Zhao

**Affiliations:** 1 Renal Division, Department of Medicine, Peking University First Hospital, Beijing, China.; 2 Peking University Institute of Nephrology, Beijing, China.; 3 Key Laboratory of Renal Disease, Ministry of Health of China, Beijing, China.; 4 Key Laboratory of Chronic Kidney Disease Prevention and Treatment (Peking University), Ministry of Education, Beijing, China.; 5 Peking-Tsinghua Center for Life Sciences, Beijing, China.

**Keywords:** Immunology, Nephrology, Complement

## Abstract

Accumulating evidence suggests the pathogenic role of immunity and metabolism in diabetic kidney disease (DKD). Herein, we aimed to investigate the effect of complement factor B (CFB) on lipid metabolism in the development of DKD. We found that in patients with diabetic nephropathy, the staining of Bb, CFB, C3a, C5a, and C5b-9 was markedly elevated in renal tubulointerstitium. *Cfb*-knockout diabetic mice had substantially milder tubulointerstitial injury and less ceramide biosynthesis. The in vitro study demonstrated that cytokine secretion, endoplasmic reticulum stress, oxidative stress, and cell apoptosis were ameliorated in HK-2 cells transfected with siRNA of *CFB* under high-glucose conditions. Exogenous ceramide supplementation attenuated the protective effect of *CFB* knockdown in HK-2 cells, while inhibiting ceramide synthases (CERS) with fumonisin B1 in *CFB*-overexpressing cells rescued the cell injury. *CFB* knockdown could downregulate the expression of NF-κB p65, which initiates the transcription of *CERS3*. Furthermore, *C3* knockdown abolished CFB-mediated cytokine secretion, NF-κB signaling activation, and subsequently ceramide biosynthesis. Thus, *CFB* deficiency inhibited activation of the complement alternative pathway and attenuated kidney damage in DKD, especially tubulointerstitial injury, by inhibiting the NF-κB signaling pathway, further blocking the transcription of *CERS*, which regulates the biosynthesis of ceramide. CFB may be a promising therapeutic target of DKD.

## Graphical abstract

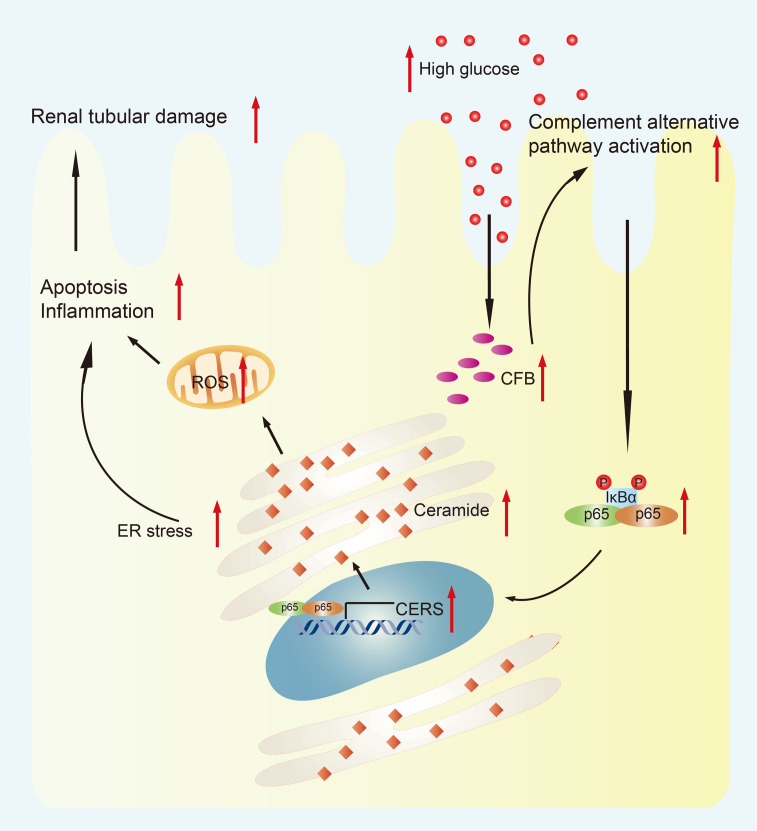

## Introduction

Diabetic kidney disease (DKD), one of the most common complications of diabetes, is becoming the leading cause of end-stage renal disease worldwide. DKD is characterized by increased urinary albumin excretion, reduced glomerular filtration rate, and distinct histopathological changes, including glomerular basement membrane thickening, mesangial expansion, nodular sclerosis, interstitial fibrosis and tubular atrophy, and interstitial infiltrates and vascular lesions (1, 2). Hyperglycemia is widely thought to lead to DKD via multiple pathways, including the production of advanced glycation end products, the aldose reductase/polyol pathway, the renin-angiotensin system, endoplasmic reticulum (ER) stress, and oxidative stress. Growing evidence suggests that the crosstalk between immunity and metabolism participates in the development and progression of DKD (3). In recent years, the influence of the complement system on lipid metabolism has aroused increasing attention (4–6).

Complement activation occurs through 3 pathways, i.e., the classical pathway, lectin pathway, and alternative pathway (AP). Several lines of evidence suggest that activation of the complement system plays a vital role in the pathogenesis of DKD (7–10). The AP is a positive feedback amplifier of the complement cascade, indispensable in many renal diseases, including C3 glomerulopathy and renal ischemia/reperfusion injury (11). Complement factor B (CFB) is the unique factor in AP and is essential for AP activation (12). Li et al. found that Toll-like receptor 4 (TLR4) upregulated the expression of CFB in renal tubular cells (13). Significantly less albuminuria, renal dysfunction, and renal cortical nuclear factor κB (NF-κB) activation were found in *Tlr4*-deficient streptozotocin-induced diabetic mice than wild-type (WT) diabetic mice (14). In addition, our recent study found that in patients with diabetic nephropathy (DN), both circulating and urinary levels of CFB were significantly higher than in healthy controls (HCs), and urinary levels of CFB correlated with the severity of DN (15).

Several studies suggested the regulatory role of AP on lipid metabolism (5, 6, 16). Moreover, it has been reported that CFB promotes adipocyte differentiation, maturation, and lipid droplet formation (6). C3a produced by AP is cleaved to acylation-stimulating proteins and binds C5aR2 to promote lipid storage (16). The study by Kunchithapautham et al. found that in age-related macular degeneration, oxidative stress and AP were activated by smoke exposure, resulting in ER stress–mediated lipid accumulation in retinal pigment epithelium (5). Based on the above evidence, we hypothesized that AP activation mediates lipid metabolism and, thus, plays an indispensable role in the pathogenesis of DKD.

To validate this hypothesis, we assessed the deposition of Bb, CFB, C3a, C5a, and C5b-9 in the kidneys of patients with DN and the difference in disease phenotypes between *Cfb*-knockout (*Cfb^–/–^*) diabetic mice and WT diabetic mice induced by streptozotocin (STZ) and high-fat diet (HFD). Furthermore, we explored the specific mechanism of lipid metabolism regulated by CFB in DKD.

## Results

### The complement AP is activated in renal biopsies of patients with DN.

**Figure 1:**
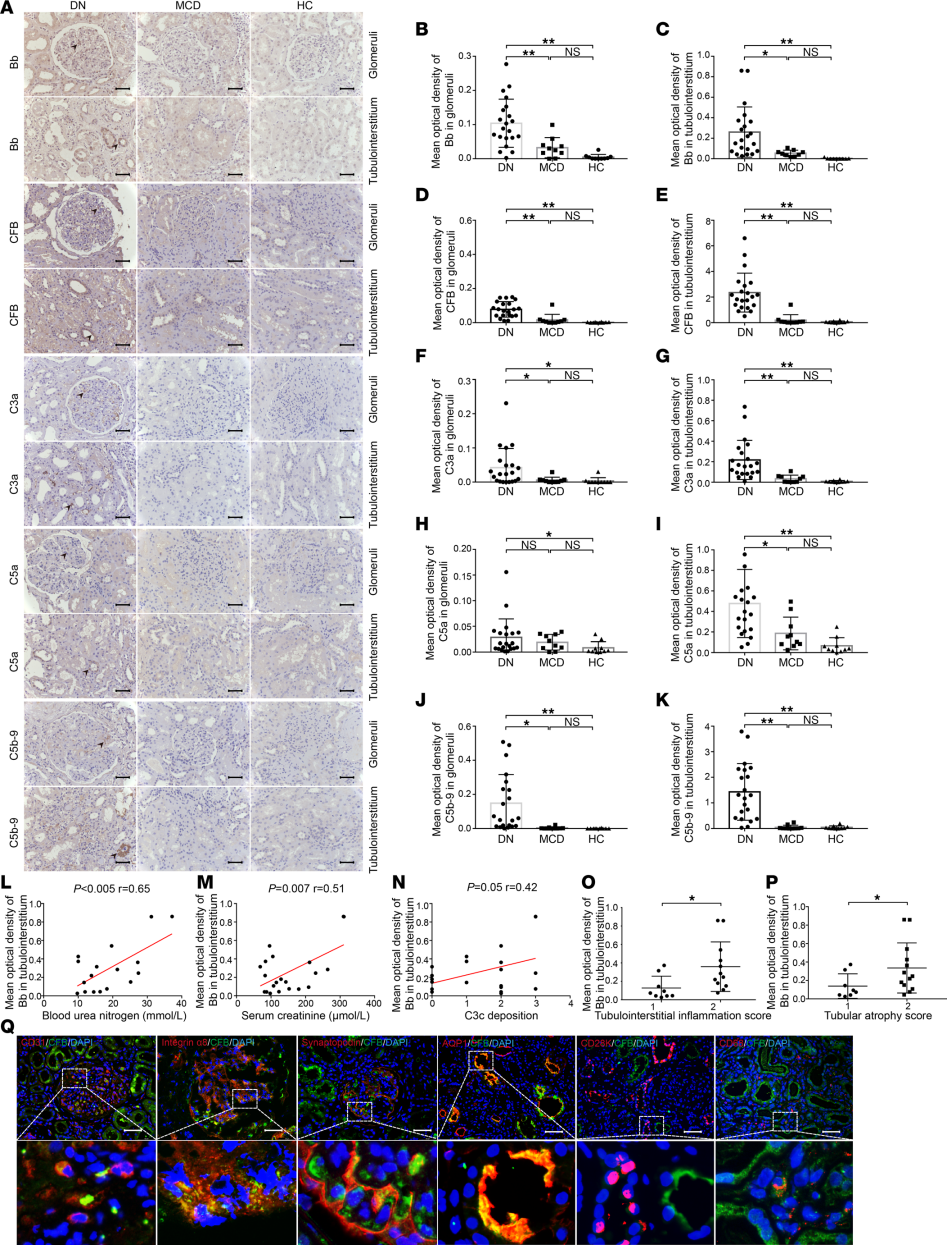
The complement AP is activated in renal biopsies of patients with DN. Immunohistochemical staining for Bb, CFB, C3a, C5a, and C5b-9 in glomeruli and tubulointerstitium (black arrow) of patients with DN (*n* = 21), patients with MCD (*n* = 10), and HCs (*n* = 10); bar = 50 μm (**A**). Semiquantitative analysis of Bb, CFB, C3a, C5a, and C5b-9 staining in glomeruli (**B**, **D**, **F**, **H**, and **J**) and tubulointerstitium (**C**, **E**, **G**, **I**, and **K**) was performed using Image-Pro Plus 6.0 software. Correlation analysis of BUN (**L**), serum creatinine (**M**), and C3c deposition (**N**) with the level of Bb deposition in tubulointerstitium of patients with DN. Comparisons of Bb deposition in tubulointerstitium between DN patients with different severities of tubulointerstitial inflammation (**O**) and tubular atrophy (**P**). Double-immunofluorescence staining of CFB (green) and renal intrinsic cells and macrophages (red); bar = 50 μm; magnified insets, original magnification, ×400 (**Q**). *P < 0.05; **P < 0.01 between groups was determined by Student’s *t* test (**O** and **P**), Pearson’s test (**N**), or Spearman’s test (**L** and **M**) as appropriate, or 1-way ANOVA (**B**–**K**). BUN, blood urea nitrogen; DN, diabetic nephropathy; HC, healthy controls; MCD, minimal change disease; MOD, mean optical density.

We investigated the deposition of Bb and CFB in renal specimens of patients with DN (*n* = 21), patients with minimal change disease (MCD; *n* = 10), and HCs (*n* = 10) by immunohistochemical staining to investigate complement AP activation in kidneys. The deposition of Bb and CFB was mainly observed in renal tubulointerstitium and, to a lesser extent, in glomeruli of DN patients. Bb and CFB depositions were significantly higher in both glomeruli and tubulointerstitium of DN patients than in MCD patients and HCs (Figure 1, A–E). Moreover, staining for downstream molecules of complement activation, including C3a, C5a, and C5b-9, was conducted. The deposition of C3a and C5b-9 was significantly higher in both glomeruli and tubulointerstitium of DN patients than in MCD patients and HCs (Figure 1, A, F, G, J, and K). Moreover, C5a deposition was significantly higher in the tubulointerstitium but not in the glomeruli in patients with DN than in patients with MCD and HCs (Figure 1, A and H–I). Bb deposition significantly correlated with C3a and C5a and marginally correlated with C5b-9 in the tubulointerstitium of patients with DN (Supplemental Figure 1, A–C; supplemental material available online with this article; https://doi.org/10.1172/jci.insight.156748DS1).

Furthermore, in patients with DN, the deposition of Bb in tubulointerstitium positively correlated with serum levels of total cholesterol (TCHO) and low-density lipoprotein (LDL) (Supplemental Figure 1, D and E), BUN, serum creatinine, and C3c deposition (Figure 1, L–N) in kidney biopsy specimens. Patients with DN with a score of 2 in tubulointerstitial inflammation and tubular atrophy had significantly higher intensity of Bb staining in tubulointerstitium than those with a score of 1 (Figure 1, O and P).

To identify the localization of CFB in the kidneys of patients with DN, double-immunofluorescence staining was performed (Figure 1Q). It was found that CFB was mainly expressed in glomerular endothelial cells, mesangial cells, podocytes, proximal tubular epithelial cells (PTECs), and macrophages but not in distal tubular epithelial cells. Furthermore, CFB and C3a were colocalized in both glomeruli and tubulointerstitium in patients with DN (Supplemental Figure 1F).

### Cfb deficiency attenuates renal damage in diabetic mice.

To assess the effect of genetic deletion of CFB on the development of DKD, *Cfb^–/–^* mice and their WT littermates were treated with STZ and HFD to induce diabetes (Supplemental Figure 2A). All diabetic mice displayed significantly lower body weight, higher levels of fasting plasma glucose (FPG), higher kidney-to-body weight ratio, and higher levels of plasma TCHO, triglyceride, and BUN compared with nondiabetic mice. *Cfb^–/–^* diabetic mice displayed slightly lower plasma triglyceride and TCHO levels than WT diabetic mice. Besides, *Cfb^–/–^* diabetic mice presented significantly lower urinary albumin-to-creatinine ratio (uACR) levels than WT diabetic mice (Table 1).

**Figure 2:**
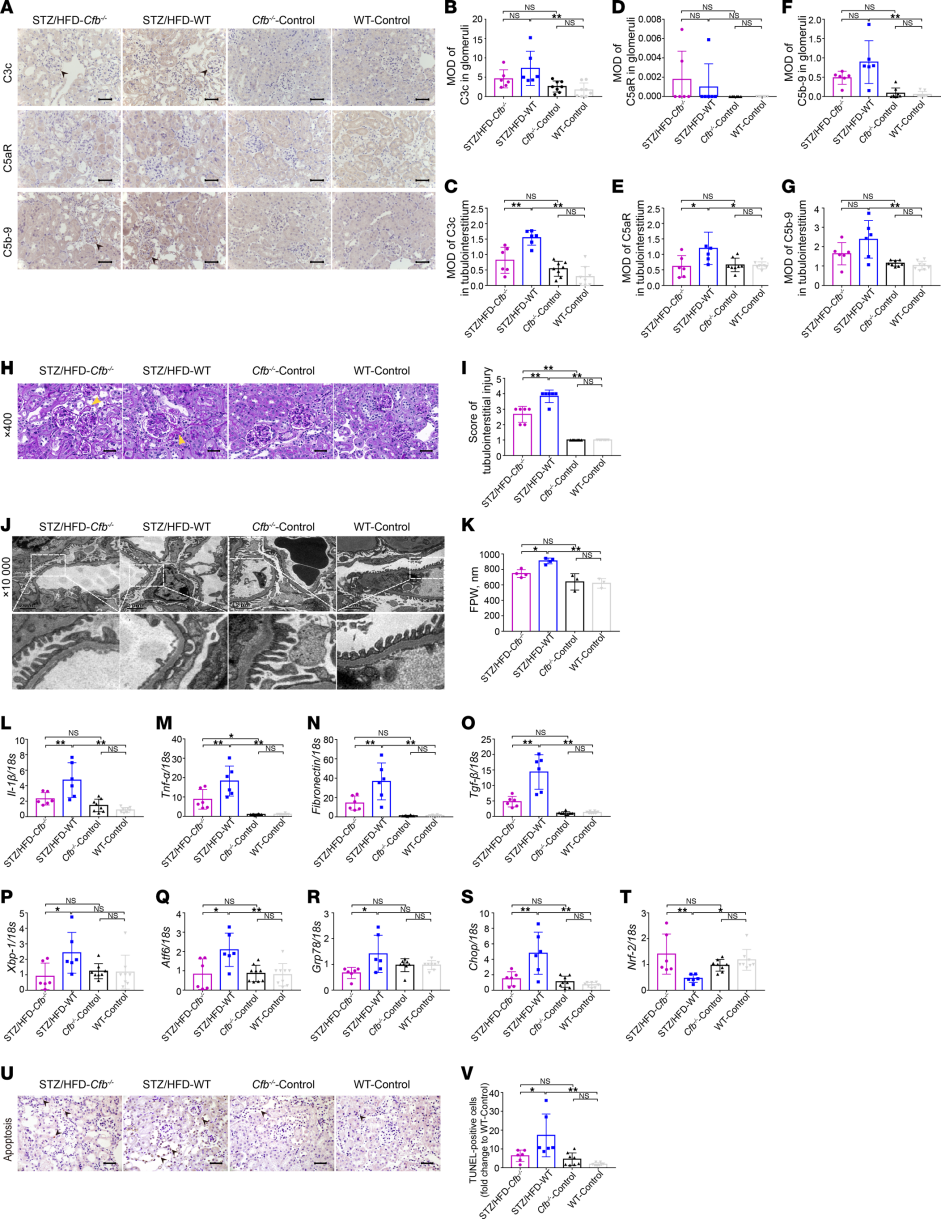
Kidney damage is alleviated in *Cfb*-deficient diabetic mice. Immunohistochemical staining for C3c, C5aR, and C5b-9 in the mouse kidney (black arrow); bar = 50 μm (**A**). Semiquantitative analysis of C3c, C5aR, and C5b-9 staining in glomeruli (**B**, **D**, and **F**) and tubulointerstitium (**C**, **E**, and **G**) was performed using Image-Pro Plus 6.0 software; *n* = 6 for diabetic groups, *n* = 8 for nondiabetic groups. We used PAS staining to represent renal tubulointerstitial damage (yellow arrow) of *Cfb^–/–^* diabetic mice (*n* = 6), WT diabetic mice (*n* = 6), *Cfb^–/–^* control mice (*n* = 8), and WT control mice (*n* = 8); bar = 50 μm (**H**). Semiquantitative analysis of tubulointerstitial injury among the above 4 groups (**I**). Electron microscopy images and partial enlargement images were used to show the fusion of podocyte foot; *n* = 4 for diabetic groups, *n* = 3 for nondiabetic groups, bar = 2 μm (**J**). Semiquantitative analysis of FPW (**K**). RT-qPCR was used to measure *Il-1β* (**L**), *Tnf-α* (**M**), *fibronectin* (**N**), *Tgf-β1* (**O**), *Xbp-1* (**P**), *Atf6* (**Q**), *Grp78* (**R**), *Chop* (**S**), and *Nrf-2* (**T**) mRNA expression in kidney cortex of mice; *n* = 6 for diabetic groups, *n* = 8 for nondiabetic groups. Representative photomicrographs of TUNEL staining (black arrow), bar = 50 μm (**U**). Semiquantitative analysis of apoptotic cell ratio of each group; *n* = 6 for diabetic groups, *n* = 8 for nondiabetic groups (**V**). *P < 0.05; **P < 0.01 between groups was determined by 1-way ANOVA. *Atf6*, activating transcription factor 6; *Chop*, CCAAT enhancer binding protein homologous protein; FPW, mean width of the foot processes; *Grp78*, glucose-regulated protein 78; *Il-1β*, interleukin 1β; MOD, mean optical density; *Nrf-2*, nuclear factor, erythroid 2 like 2; PAS, periodic acid–Schiff; RT-qPCR, quantitative real-time PCR; *Tgf-β1*, transforming growth factor β1; *Tnf-α*, tumor necrosis factor α; TUNEL, terminal deoxynucleotidyl transferase–mediated dUTP nick end labeling; WT, wild-type; *Xbp-1*, X-box binding protein 1.

The extent of complement activation was detected by examining the levels of C3c, C5aR, and C5b-9 deposition in kidney specimens of mice. Compared with WT diabetic mice, significantly lower C3c and C5aR levels were detected in the tubulointerstitium of *Cfb^–/–^* diabetic mice by immunohistochemical staining (Figure 2, A, C, and E). There was no significant difference in C3c, C5aR, or C5b-9 deposition in the glomeruli (Figure 2, A, B, D, and F) or C5b-9 deposition in the tubulointerstitium (Figure 2, A and G) between *Cfb^–/–^* diabetic mice and WT diabetic mice. Moreover, *Cfb^–/–^* diabetic mice displayed significantly milder tubulointerstitial injury than WT diabetic mice (Figure 2, H and I). Electron microscopy showed diffuse podocyte effacement and thickening of glomerular basement membrane (GBM) in diabetic mice compared with nondiabetic mice, while *Cfb^–/–^* diabetic mice exhibited significantly less fusion of podocyte foot compared with WT diabetic mice (Figure 2, J and K). In addition, there was no significant difference in glomerular hypertrophy, mesangial matrix expansion, or thickening of GBM between *Cfb^–/–^* diabetic mice and WT diabetic mice (Supplemental Figure 2, B–D).

We used the kidney cortex of mice to assess the mRNA levels of proinflammatory and profibrotic cytokines. We found that *Cfb^–/–^* diabetic mice had significantly lower levels of proinflammatory factors (*Il-1β* and *Tnf-α*) and profibrotic factors (*fibronectin* and *Tgf-β1*) than the WT diabetic group (Figure 2, L–O).

To assess ER stress and oxidative stress, we examined 3 activating pathways of ER stress and the main antioxidant genes. Compared with WT diabetic mice, the mRNA levels of *Xbp-1*, *Atf6*, *Grp78*, and *Chop* were significantly downregulated, and *Nrf-2* was significantly upregulated in *Cfb^–/–^* diabetic mice (Figure 2, P–T). Thus, *Cfb* knockout could decrease ER stress and oxidative stress levels in diabetic mice. In addition, *Cfb^–/–^* diabetic mice had lower levels of apoptotic cells than WT diabetic mice (Figure 2, U and V), and the apoptotic cells were mainly located in the tubulointerstitium.

The above results suggested that both glomerular and tubulointerstitial injuries were alleviated in *Cfb^–/–^* diabetic mice compared with WT diabetic mice, and the alleviation of tubulointerstitial injury was more pronounced. In addition to the improvement of inflammation, we found that oxidative stress, ER stress, and apoptosis were alleviated in *Cfb^–/–^* diabetic mice.

### Cfb deficiency decreases the biosynthesis of ceramide in diabetic mice.

**Figure 3:**
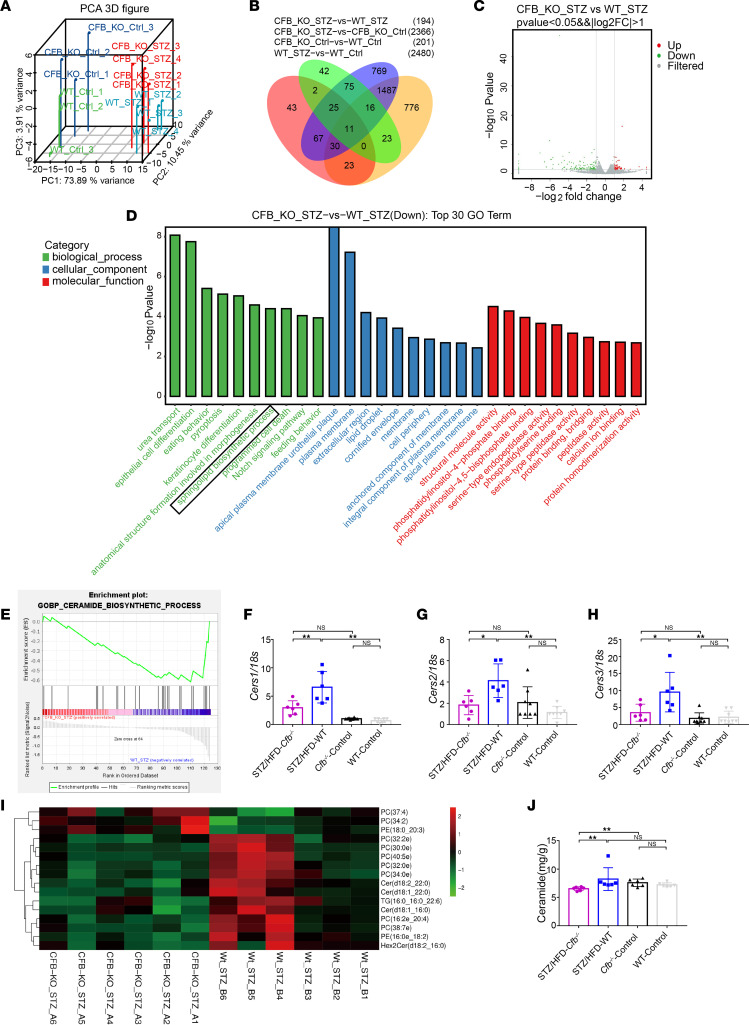
The process of ceramide biosynthesis was downregulated in *Cfb*-deficient diabetic mice. PCA was conducted with gene expression level to illustrate the distribution and repeatability of each sample; *n* = 4 for diabetic groups, *n* = 3 for nondiabetic groups (**A**). The number of common and unique DEGs was analyzed (**B**). We used a volcano map to present the upregulated or downregulated genes between *Cfb^–/–^* diabetic mice and WT diabetic mice (**C**). Compared with WT diabetic mice, GO enrichment was conducted with the top 30 terms of downregulated categories of *Cfb^–/–^* diabetic mice (**D**). GSEA of transcriptomics was conducted from *Cfb^–/–^* diabetic mice compared with WT diabetic mice. This targeted GSEA of sphingolipid synthesis signaling was performed in addition to and separately from a comprehensive transcriptomics analysis (**E**). RT-qPCR was used to measure and verify the mRNA expression of *Cers* in the kidney cortex of mice; *n* = 6 for diabetic groups, *n* = 8 for nondiabetic groups (**F**–**H**). Untargeted lipidomics and the quantification of ceramide were performed to illustrate the downregulated ceramide biosynthesis in *Cfb^–/–^* diabetic mice; *n* = 6/group (**I** and **J**). *P < 0.05; **P < 0.01 between groups was determined by 1-way ANOVA. *Cers*, ceramide synthases; GSEA, gene set enrichment analysis; PCA, principal component analysis; RT-qPCR, quantitative real-time PCR; WT, wild-type.

To explore the underlying mechanism of CFB in the development of DKD, we performed transcriptomics for global gene sequencing. Principal component analysis (PCA) was conducted with gene expression levels to illustrate the distribution and repeatability of each sample in different groups (Figure 3A). The number of differentially expressed genes (DEGs) in different groups is shown in Figure 3B. *Cfb^–/–^* diabetic mice had 194 DEGs (based on the criteria of *P* < 0.05 and fold change > 2.0 or < 0.5) compared with WT diabetic mice, including 59 upregulated and 135 downregulated genes (Figure 3C). Compared with WT diabetic mice, Gene Ontology (GO) enrichment analysis was conducted with the top 30 terms of downregulated categories of *Cfb^–/–^* diabetic mice, including the sphingolipid synthesis process and programmed cell death (Figure 3D). In addition, gene set enrichment analysis (GSEA) specifically for sphingolipid synthesis signaling on data from transcriptomics was performed. Compared with WT diabetic mice, genes involved in the ceramide biosynthesis process were significantly downregulated in *Cfb^–/–^* diabetic mice (FDR = 0.085; Figure 3E).

Based on the transcriptomic assay of DEGs, we used RT-qPCR to verify and quantify the main enzymes responsible for ceramide biosynthesis, including 6 subtypes of ceramide synthases (*Cers*). Of those, in *Cfb^–/–^* diabetic mice, the gene expressions of *Cers1*, *Cers2*, and *Cers3*, which are responsible for ceramide biosynthesis, were significantly lower than in WT diabetic mice (Figure 3, F–H).

Next, we used untargeted lipidomics to examine the change in lipid species. Phosphatidylcholine, phosphatidylethanolamine, ceramide, triglyceride, and Hex2Cer were altered upon *CFB* deficiency. Among sphingolipids, Cer (d18:2_22:0), Cer (d18:1_22:0), and Cer (d18:1_16:0) (Figure 3I), and the quantity of total ceramide, were significantly decreased in *Cfb^–/–^* diabetic mice (Figure 3J), compared with WT diabetic mice. These results substantiated the downregulation of the ceramide biosynthesis process upon *Cfb* knockout.

### CFB knockdown alleviates PTEC damage and ceramide biosynthesis under high-glucose stimulation.

Since our above results demonstrated that CFB was mainly expressed in the tubulointerstitium of patients with DN, we explored the role of CFB in PTECs at the cellular level. We found that high-glucose (HG) treatment to HK-2 cells significantly increased the expression of *CFB*, *C3*, *IL-1β*, *IL-6*, *CCL2*, *TNF-α*, *TGF-β1*, and *fibronectin*, compared with the osmotic control (Supplemental Figure 3, A–H).

Then we transfected the siRNA of *CFB* (si*CFB*) under the above conditions. The transfection efficiency was 70% to 80% (Supplemental Figure 4A), and the protein levels of CFB were also significantly decreased (Supplemental Figure 4, B and C). Knocking down *CFB* could significantly downregulate the level of C3a in the supernatant of HK-2 cells under HG stimulation (Supplemental Figure 4D), suggesting that the activation of complement AP was attenuated.

**Figure 4:**
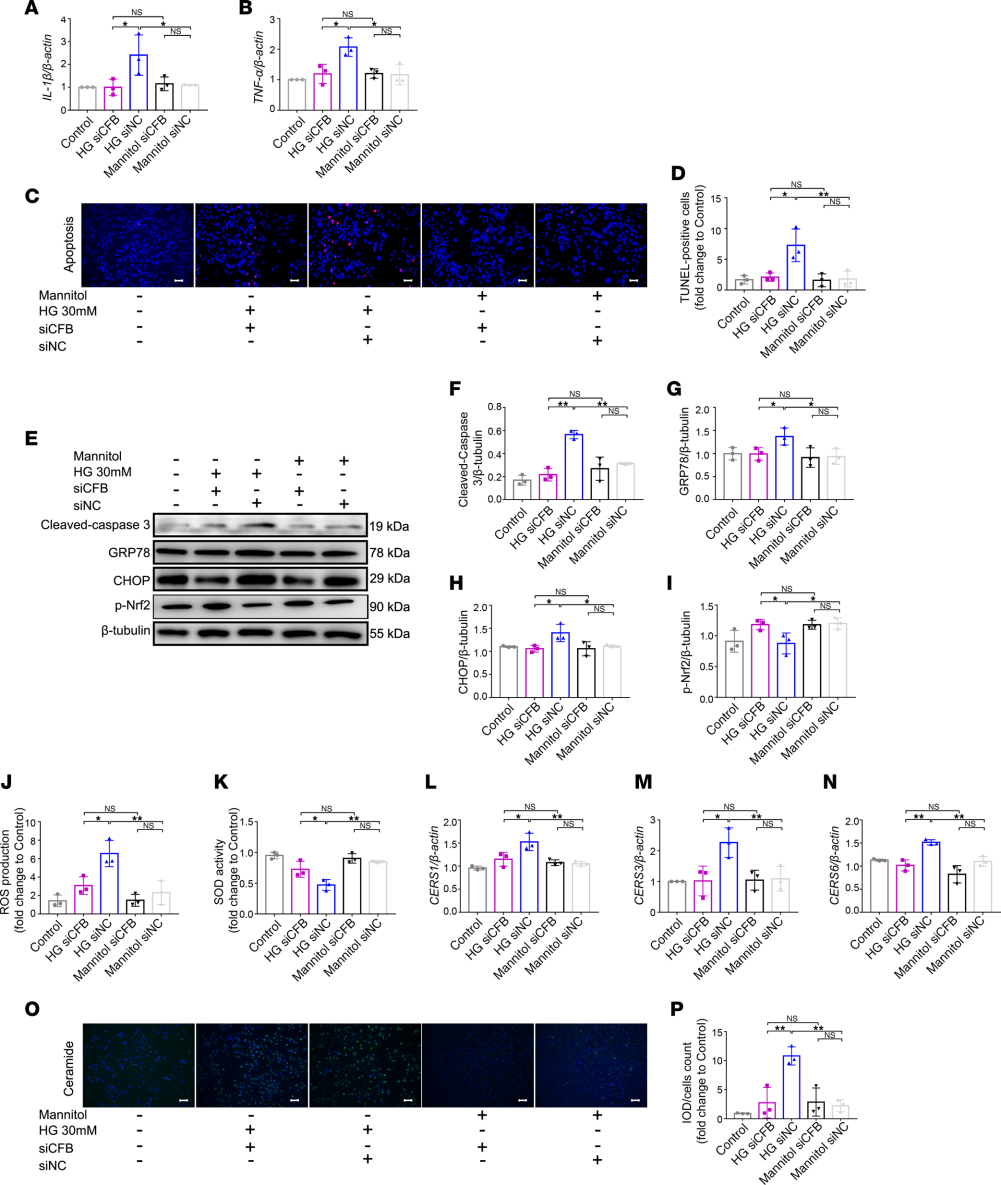
*CFB* knockdown alleviates PTEC damage and ceramide biosynthesis under HG condition. RT-qPCR was used to measure the expression of *IL-1β* (*n* = 3/group) and *TNF-α* (*n* = 3/group) under HG conditions in si*CFB* HK-2 cells (**A** and **B**). Representative photomicrographs of TUNEL staining, bar = 50 μm (**C**). Semiquantitative analysis of apoptotic cell ratio of each group, *n* = 3/group (**D**). Western blot was used to measure the expression level of cleaved caspase-3, GRP78, CHOP, and p-Nrf2 (**E**), and the ratios of the above protein to β-tubulin were quantified; *n* = 3/group (**F**–**I**). The levels of intracellular ROS production (**J**) and total SOD activity (**K**) were measured among 5 groups; *n* = 3/group. RT-qPCR was used to measure the mRNA expression of *CERS* (**L**–**N**); *n* = 3/group. IF staining was used to illustrate the quantity of ceramide bar = 50 μm, *n* = 3/group (**O**), and semiquantitative analysis of ceramide staining was performed (**P**). *P < 0.05; **P < 0.01 between groups was determined by 1-way ANOVA. CERS, ceramide synthases; CHOP, CCAAT enhancer binding protein homologous protein; GRP78, glucose-regulated protein 78; IF, immunofluorescence; IL-1β, interleukin 1β; p-Nrf-2, phosphorylated nuclear factor, erythroid 2 like 2; PTECs, proximal tubular epithelial cells; RT-qPCR, quantitative real-time PCR; ROS, reactive oxygen species; SOD, superoxide dismutase; TNF-α, tumor necrosis factor α; TUNEL, terminal deoxynucleotidyl transferase–mediated dUTP nick end labeling.

Compared with HK-2 cells treated with HG and siRNA of negative control (si*NC*+HG group), si*CFB* could significantly downregulate the expression of *IL-1β* and *TNF-α* (Figure 4, A and B). The apoptosis of HK-2 cells was detected with the TUNEL assay (Figure 4, C and D), and the protein level of cleaved caspase-3 (Figure 4, E and F) was measured. Compared with the si*NC*+HG group, they were significantly decreased in si*CFB* HK-2 cells stimulated with HG.

We also detected the extent of ER stress and oxidative stress in HK-2 cells treated with HG and transfected with si*CFB*. It was found that GRP78 and CHOP, 2 indicators of the activation of ER stress, were significantly reduced, and phosphorylated Nrf-2 (p-Nrf2), an indicator of antioxidant ability, was significantly increased in the si*CFB*+HG group (Figure 4, E and G–I). Intracellular reactive oxygen species (ROS) production and total superoxide dismutase (SOD) activity were significantly improved in the si*CFB*+HG group (Figure 4, J and K) compared with the si*NC*+HG group.

In addition, we verified the results acquired from transcriptomics and lipidomics of the animal model. We examined the expression of *Cers* in mouse kidney tubular epithelium (TCMK-1) cells transfected with si*Cfb*. The efficiency of knockdown was around 50% (Supplemental Figure 2E). RT-qPCR demonstrated a significant decrease of *Cers1*, *Cers2*, and *Cers3* in TCMK-1 cells transfected with si*Cfb* under HG stimulation (Supplemental Figure 2, F–H). Compared with the si*NC*+HG group, the mRNA levels of *CERS1*, *CERS3*, and *CERS6* were significantly reduced in HK-2 cells in the si*CFB*+HG group (Figure 4, L–N). Ceramide staining was also significantly decreased in HK-2 cells in the si*CFB*+HG group (Figure 4, O and P).

### CFB knockdown alleviates PTEC damage via downregulating ceramide levels under HG conditions.

To illustrate the role of ceramide on CFB-mediated PTEC damage under HG conditions, we supplemented ceramide exogenously in si*CFB*-transfected HK-2 cells. The efficiency of transfection was 70%–80% (Supplemental Figure 4E). HK-2 cells were treated with C2-ceramide (C2-Cer; 20 μmol/L) or its inactive form, C2-dihydroceramide, as the control.

**Figure 5:**
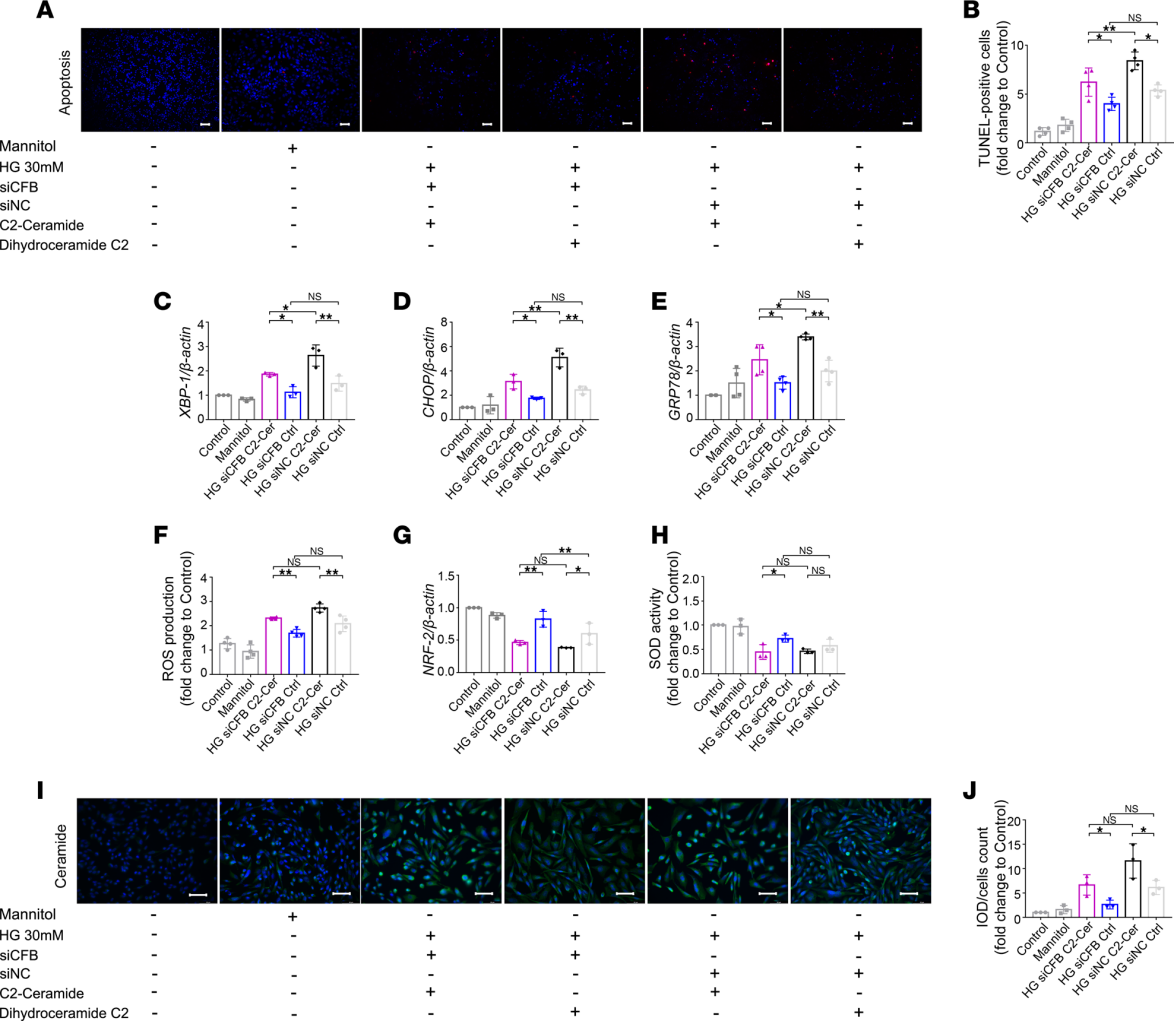
*CFB* knockdown alleviates PTEC damage via downregulating ceramide under HG conditions. Representative photomicrographs of TUNEL staining, bar = 50 μm, *n* = 4/group (**A**). Semiquantitative analysis of apoptotic cell ratio of each group (**B**). RT-qPCR was used to measure the expression level of *XBP-1* (**C**, *n* = 3/group), *CHOP* (**D**, *n* = 3/group), *GRP78* (**E**, *n* = 4/group), and *NRF-2* (**G**, *n* = 3/group). The levels of intracellular ROS production (**F**, *n* = 4/group) and total SOD activity (**H**, *n* = 3/group) were measured. IF staining was used to illustrate the quantity of ceramide; bar = 50 μm (**I**), and semiquantitative analysis of ceramide staining was calculated, *n* = 3/group (**J**). *P < 0.05; **P < 0.01 between groups was determined by 1-way ANOVA. CHOP, CCAAT enhancer binding protein homologous protein; GRP78, glucose-regulated protein 78; IF, immunofluorescence; IOD, integrated optical density; NRF-2, nuclear factor, erythroid 2 like 2; PTECs, proximal tubular epithelial cells; RT-qPCR, quantitative real-time PCR; ROS, reactive oxygen species; SOD, superoxide dismutase; TUNEL, terminal deoxynucleotidyl transferase–mediated dUTP nick end labeling; XBP-1, X-box binding protein 1.

Compared with the si*CFB*/C2-dihydroceramide+HG group, the apoptotic cells stained by the TUNEL assay were significantly increased in the si*CFB*/C2-Cer+HG group (Figure 5, A and B). ER stress and oxidative stress were exacerbated when si*CFB* HK-2 cells were treated with C2-Cer. The mRNA levels of *XBP-1*, *CHOP*, and *GRP78* were significantly increased in the si*CFB*/C2-Cer+HG group (Figure 5, C–E). The intracellular ROS production was significantly elevated (Figure 5F); the mRNA level of *NRF-2* and the total intracellular SOD were significantly reduced in the si*CFB*/C2-Cer+HG group (Figure 5, G and H). Furthermore, compared with the si*CFB*/C2-dihydroceramide+HG group, the ceramide staining was significantly higher in the si*CFB*/C2-Cer+HG group (Figure 5, I and J).

### Fumonisin B1 can rescue CFB-mediated PTEC damage via downregulating ceramide synthases.

**Figure 6:**
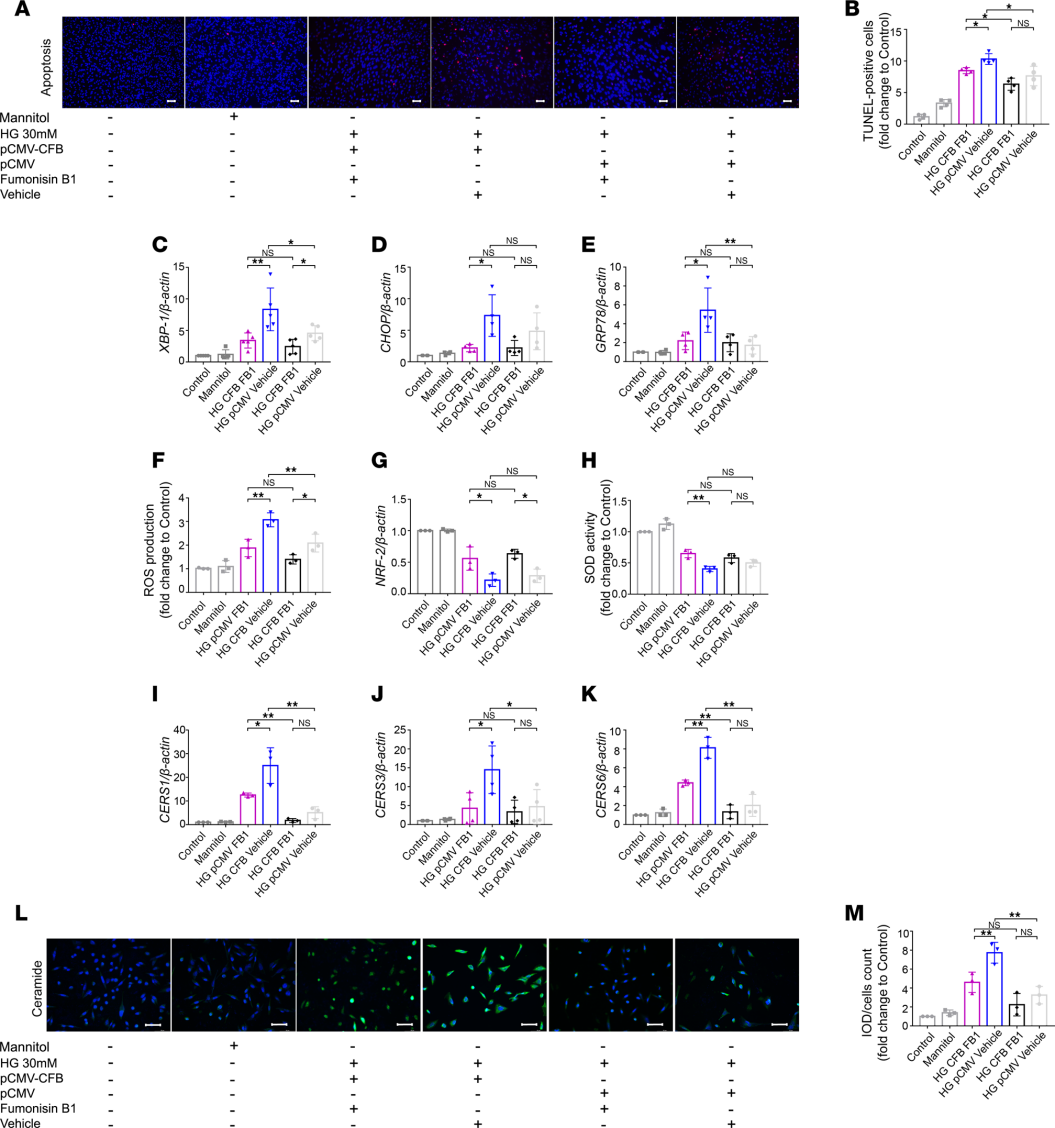
FB1 can rescue the CFB-mediated PTEC damage by downregulating CERS. Representative photomicrographs of TUNEL staining; bar = 50 μm, *n* = 4/group (**A**). Semiquantitative analysis of apoptotic cell ratio of each group (**B**). RT-qPCR was used to measure the expression level of *XBP-1* (**C**, *n* = 6/group), *CHOP* (**D**, *n* = 4/group), *GRP78* (**E**, *n* = 4/group) and *NRF-2* (**G**, *n* = 3/group). The levels of intracellular ROS production (**F**) and total SOD activity (**H**) were measured; *n* = 3/group. RT-qPCR was used to measure the expression of *CERS1* (**I**, *n* = 3/group), *CERS3* (**J**, *n* = 4/group), and *CERS6* (**K**, *n* = 3/group). IF staining was used to illustrate the quantity of ceramide, bar = 50 μm (**L**), and semiquantitative analysis of ceramide staining was calculated, *n* = 3/group (**M**). *P < 0.05; **P < 0.01 between groups was determined by 1-way ANOVA. CERS, ceramide synthases; CHOP, CCAAT enhancer binding protein homologous protein; FB1, fumonisin 1; GRP78, glucose-regulated protein 78; IF, immunofluorescence; NRF-2, nuclear factor, erythroid 2 like 2; PTECs, proximal tubular epithelial cells; RT-qPCR, quantitative real-time PCR; ROS, reactive oxygen species; SOD, superoxide dismutase; TUNEL, terminal deoxynucleotidyl transferase–mediated dUTP nick end labeling; XBP-1, X-box binding protein 1.

The above results from transcriptomics and lipidomics suggested that the decrease of ceramide may be regulated by lower expression of ceramide synthases in *Cfb^–/–^* diabetic mice compared with WT diabetic mice. To investigate whether CFB mediates PTEC damage via upregulation of ceramide synthases, HK-2 cells were incubated with fumonisin B1 (FB1; 20 μmol/L; dissolved in H_2_O), a mycotoxin that inhibits ceramide synthases, and were transfected with the plasmid of *CFB* (pCMV-*CFB*) for overexpression. First, we verified the efficiency of overexpression of *CFB* at the mRNA level, and a 2- to 3-fold increase was observed (Supplemental Figure 4F). Overexpression of *CFB* could significantly upregulate the level of C3a in the supernatant of HK-2 cells under HG stimulation (Supplemental Figure 4G), suggesting that the activation of complement AP was aggravated. Compared with the pCMV-*CFB*/vehicle+HG group, the staining of apoptotic cells was significantly decreased in *CFB*-overexpressing HK-2 cells treated with HG and FB1 (Figure 6, A and B).

ER stress and oxidative stress were alleviated in *CFB*-overexpressing HK-2 cells treated with HG and FB1. Compared with the pCMV-*CFB*/vehicle+HG group, *XBP-1*, *CHOP*, and *GRP78* were significantly reduced in the pCMV-*CFB*/FB1+HG group (Figure 6, C–E). The intracellular ROS production was significantly reduced (Figure 6F), while *NRF-2* and total SOD were significantly increased in the pCMV-*CFB*/FB1+HG group (Figure 6, G and H).

In addition, compared with the pCMV-*CFB*/vehicle+HG group, the mRNA levels of *CERS1*, *CERS3*, and *CERS6* were significantly decreased (Figure 6, I–K), and ceramide staining was significantly decreased (Figure 6, L and M) in the pCMV-*CFB*/FB1+HG group.

### CFB mediates NF-κB p65 translocation in PTECs, and NF-κB p65 initiates the transcription of CERS3.

**Figure 7:**
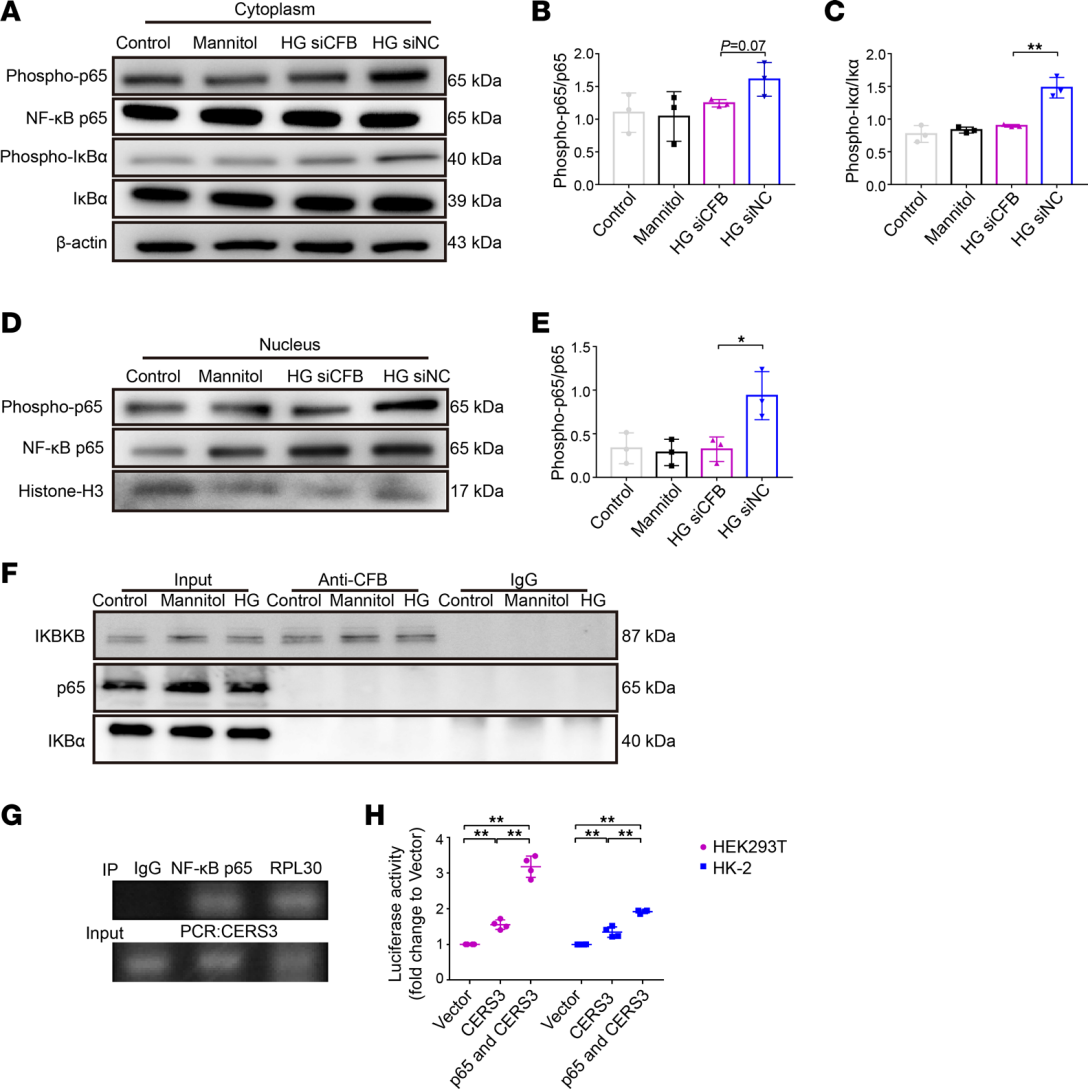
CFB mediates NF-κB p65 translocation in PTECs, and NF-κB p65 can directly bind to the promoter of *CERS3*. Western blot was used to measure the expression level of p-p65, NF-κB p65, p-IκBα, IκBα, and β-actin in the cytoplasm (**A**). The ratios of p-p65/p65 (**B**) and p-IκBα/IκBα (**C**) in the cytoplasm were quantified, *n* = 3/group. Western blot was used to measure the expression level of p-p65, NF-κB p65, and histone H3 (**D**), and the ratio of p-p65/p65 (**E**) in the nucleus was quantified, *n* = 3/group. Co-immunoprecipitation of CFB and NF-κB p65, IκBα, and IKBKB in HK-2 cells (**F**). Total protein lysates of cells were immunoprecipitated using an antibody against CFB and immunoblotting against NF-κB p65, IκBα, and IKBKB. Chromatin immunoprecipitation analysis of HK-2 cells was performed with an anti–NF-κB p65 antibody and the primers for the putative region of the *CERS3* promoter (**G**). Luciferase reporter vectors with *CERS3* promoter were cotransfected with the vector expressing NF-κB p65 into HEK293T and HK-2 cells, and luciferase reporter analysis was performed, *n* = 3/group (**H**). *P < 0.05; **P < 0.01 between groups was determined by 1-way ANOVA. CERS3, ceramide synthase 3; IKBKB, inhibitor of NF-κB kinase subunit B; phospho-, phosphorylated; PTECs, proximal tubular epithelial cells.

Next, we examined whether *CFB* deficiency protected PTECs from HG stimulation via inhibiting NF-κB p65 signaling. As shown in Figure 7A, compared with the si*NC*+HG group, the expression of p-p65/p65 in the cytoplasm marginally decreased, and p-IκBα/IκBα in the cytoplasm significantly decreased, in the si*CFB*+HG group (Figure 7, B and C). This suggested that the NF-κB p65 signaling pathway was downregulated when *CFB* was knocked down under HG conditions. Furthermore, we examined the expression of p-p65/p65 in the nucleus. HG stimulation promoted nuclear p-p65 expression, while *CFB* knockdown decreased the p-p65/p65 expression in the nucleus (Figure 7, D and E), indicating the blockage of p-p65 translocation from the cytoplasm to the nucleus. These results suggested that CFB mediated NF-κB p65 signaling in PTECs in the context of diabetes.

Furthermore, we performed co-immunoprecipitation (Co-IP) of CFB with NF-κB p65 and IκBα in HK-2 cells. The results showed that CFB did not bind NF-κB p65 or IκBα, suggesting that CFB indirectly regulates NF-κB p65 (Figure 7F). However, CFB could directly bind IKBKB (Figure 7F), suggesting CFB acts upstream of NF-κB signaling.

Considering the change of CERS in the results of transcriptomic analysis and cellular experiments, we further explored the regulatory mechanism of CERS expression. Using the online bioinformatics tool Promo alggen (Transfac 8.3), we found that NF-κB p65 is a potential transcription factor of *CERS3*, and the binding site was predicted within the promoter of *CERS3*. Exploring the potential binding site by chromatin immunoprecipitation (ChIP) analysis with an anti–NF-κB p65 antibody revealed that NF-κB p65 bound specifically to the promoter of *CERS3* in HK-2 cells, and the sequence was from –37 to –49 bp (Figure 7G). The luciferase activity of HEK293T and HK-2 cells transfected with the *CERS3* reporter plasmid was increased after overexpression of NF-κB p65 (Figure 7H). Collectively, these results suggested that CFB is essential for cytoplasmic to nuclear translocation of NF-κB p65, and NF-κB p65 in the nucleus further initiates the transcription of *CERS3*.

### C3 knockdown abolishes CFB-mediated cytokine secretion, ceramide biosynthesis, and NF-κB signaling in PTECs.

**Figure 8:**
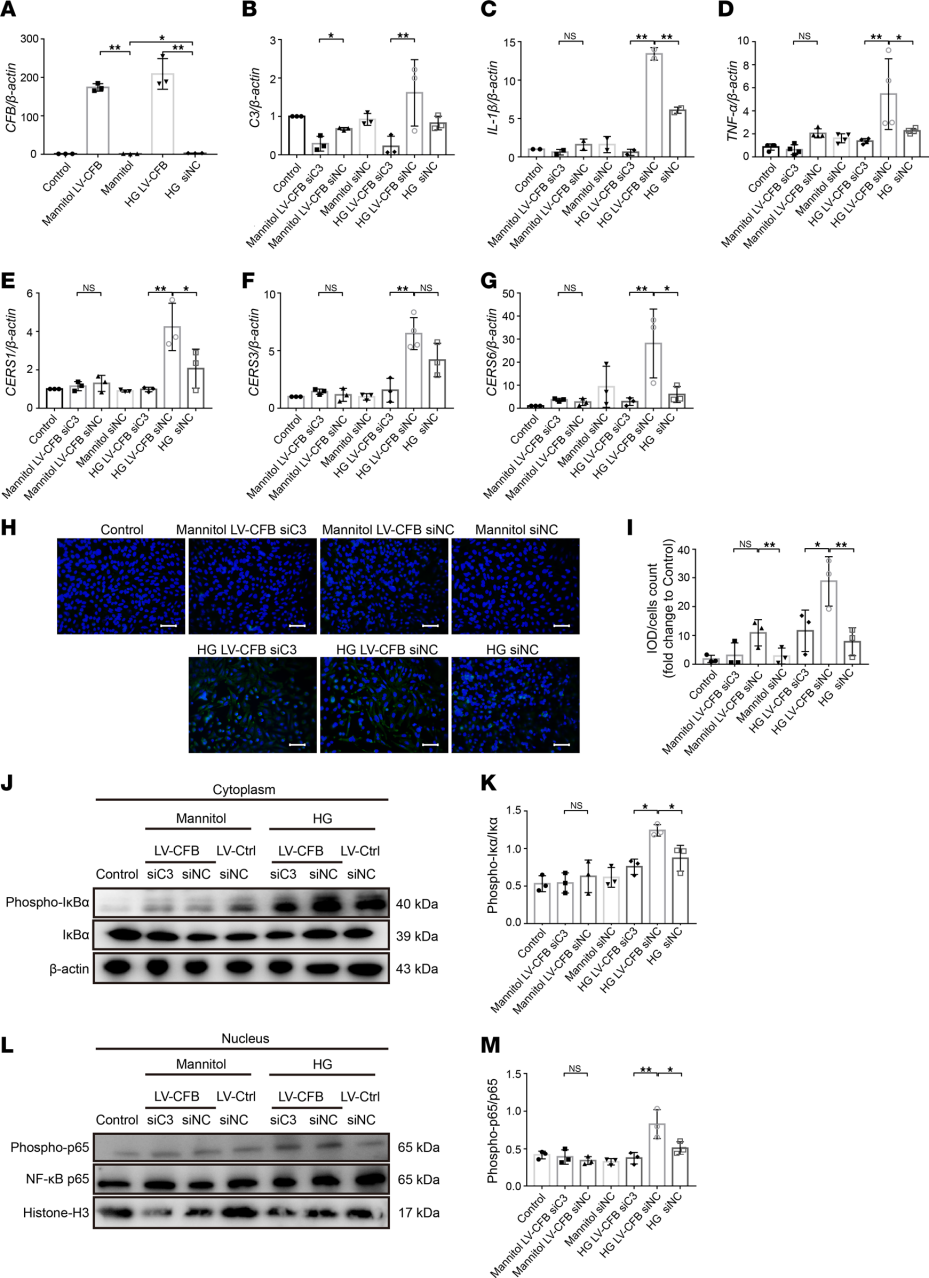
*C3* knockdown abolishes CFB-mediated cytokine secretion, ceramide biosynthesis, and NF-κB signaling in PTECs. RT-qPCR was used to measure the expression level of *CFB* (**A**, *n* = 3/group), *C3* (**B**, *n* = 3/group), *IL-1β* (**C**, *n* = 3/group), *TNF-α* (**D**, *n* = 4/group), *CERS1* (**E**, *n* = 3/group), *CERS3* (**F**, *n* = 4/group), and *CERS6* (**G**, *n* = 3/group). IF staining was used to illustrate the quantity of ceramide, bar = 50 μm (**H**), and semiquantitative analysis of ceramide staining was calculated, *n* = 3/group (**I**). Western blot was used to measure the expression level of p-p65, NF-κB p65, p-IκBα, IκBα, and β-actin in the cytoplasm (**J**), and the ratio of p-IκBα/IκBα (**K**) in the cytoplasm was quantified, *n* = 3/group. Western blot was used to measure the expression level of p-p65, NF-κB p65, and histone H3 (**L**), and the ratio of p-p65/p65 (**M**) in the nucleus was quantified, *n* = 3/group. *P < 0.05; **P < 0.01 between groups was determined by 1-way ANOVA. CERS, ceramide synthases; IF, immunofluorescence; IL-1β, interleukin 1β; phospho-, phosphorylated; PTECs, proximal tubular epithelial cells; RT-qPCR, quantitative real-time PCR; TNF-α, tumor necrosis factor α.

We found that CFB could upregulate NF-κB signaling, which stimulates the biosynthesis of ceramide. Then we examined the regulatory effect of CFB on NF-κB signaling and ceramide biosynthesis via complement AP activation. HK-2 cells were transfected with lentivirus of *CFB* (LV-*CFB*) for overexpression and si*C3* for blocking the complement activation simultaneously. First, we verified the efficiency of *CFB* overexpression and *C3* knockdown at the mRNA level (Figure 8, A and B). Overexpression of *CFB* significantly increased the mRNA levels of *IL-1β* and *TNF-α* under HG stimulation. Compared with the LV-*CFB*+si*NC*+HG group, the expression of *IL-1β* and *TNF-α* was significantly decreased in the LV-*CFB*+si*C3*+HG group (Figure 8, C and D). Meanwhile, the expression of *CERS1*, *CERS3*, and *CERS6* (Figure 8, E–G) and ceramide staining (Figure 8, H and I) were significantly lower in the LV-*CFB*+si*C3*+HG group than in the LV-*CFB*+si*NC*+HG group. Furthermore, compared with the LV-*CFB*+si*NC*+HG group, the expressions of p-IκBα/IκBα (Figure 8, J and K) in the cytoplasm and p-p65/p65 (Figure 8, L and M) in the nucleus were significantly decreased in the LV-*CFB*+si*C3*+HG group.

The above results suggested that the effects of CFB on cytokine secretion, ceramide biosynthesis, and NF-κB signaling were mediated by complement AP activation.

## Discussion

Several lines of evidence suggested the important role of the complement system in the pathogenesis of DKD (17–19). However, the exact pathogenic role of complement activation in DKD is not fully clear yet. Some clues indicated that activation of the AP participates in the development of DKD (9). A previous transcriptome analysis found that the mRNA level of *CFB* is significantly higher in the glomeruli and renal tubules of patients with DKD (9). Our recent study found that the urinary levels of CFB correlate with the severity of DKD (15).

In the current study, patients with DN had higher levels of Bb deposition in kidneys than patients with MCD and HCs. In agreement with our results, Welch et al. found that the level of locally synthesized CFB in PTECs roughly corresponded to the location and intensity of patchy tubular atrophy and interstitial inflammation of DKD (20). They also found that the expression of CFB and C3 had similar distribution in renal tubules rather than in the glomeruli, and the activation of AP caused peritubular inflammation and edema to fibrosis and tubular atrophy (20). Herein, we found that the deposition of Bb in tubulointerstitium correlated with the severity of DKD, including BUN and serum creatinine levels, and C3c deposition, tubulointerstitial inflammation, and tubular atrophy in renal histology.

The importance of tubulointerstitial injuries in DKD has been increasingly recognized in recent years, accompanied by a shift of the concept from classic DN to DKD (21, 22). Growing evidence suggests that the proximal renal tubules are implicated in the development and progression of DKD (23). Furthermore, several studies demonstrated that the degree of renal dysfunction is more closely associated with chronic tubulointerstitial injury than glomerular injury (24, 25). Combined with the findings of our study, delaying tubulointerstitial damage by inhibiting CFB in DKD is of interest.

We found that diabetic mice deficient in *Cfb* were substantially protected from functional and morphological lesions of DKD; in particular, the alleviation of tubulointerstitial injury was more prominent than glomerular injury. These results suggested that CFB plays an important role in the pathogenesis of DKD, especially in tubulointerstitial injury. Then we corroborated that the proinflammatory and profibrotic cytokines and apoptosis in the kidney cortex were reduced in *Cfb^–/–^* diabetic mice compared with WT diabetic mice. ER stress and oxidative stress are closely interconnected and eventually lead to cell apoptosis. ER stress occurs when the folding capability of the ER fails to accommodate the load of unfolded proteins (26). The oxidative stress could be aggravated by the sustained generation of ROS and improved by antioxidants such as Nrf-2 and SOD. Excessive ROS produced by oxidative stress was reported to promote cell injury and apoptosis (27). As expected, we found that both ER stress and oxidative stress were also alleviated in *Cfb^–/–^* diabetic mice.

Next, we explored the role of CFB in the pathogenesis of DKD. Previous studies demonstrated the regulatory role of the complement system on lipid metabolism in various metabolic diseases (4–6). In the current study, the lipidomic analysis revealed a potential role of CFB on lipid metabolism, especially sphingolipid synthesis. In *Cfb^–/–^* diabetic mice, total ceramide quantification and several species of ceramide were decreased, including Cer (d18:2_22:0), Cer (d18:1_22:0), and Cer (d18:1_16:0), as compared with WT diabetic mice. Furthermore, genes of ceramide biosynthesis were enriched by transcriptomic analysis. Based on the omics results, we used RT-qPCR to verify the DEGs in the cortex of diabetic mice and TCMK-1 cells; the results were in accordance with the lipidomic and transcriptomic analyses.

It was reported that in patients with DKD, urinary concentrations of Cer (d18:1/16:0) and Cer (d18:1/22:0) are increased and correlated with urinary levels of albumin and *N*-acetyl-β-d-glucosaminidase (28). Ceramide is at the center of sphingolipid metabolism and is formed via 3 main pathways: de novo synthesis, hydrolysis of sphingomyelin, and salvage of complex sphingolipids. Ceramide contributes to inflammation, cell membrane dynamics, cell growth, and apoptosis (29). Ceramide is highly abundant in the kidney and is actively involved in the pathogenesis of diabetes and its complications (30–34). Furthermore, we found that *Cers1*, *Cers2*, *Cers3*, and ceramide were reduced in *Cfb^–/–^* diabetic mice, suggesting the decrease in ceramide levels may result from the decrease of ceramide synthases.

Our cellular experiments found that proinflammatory cytokines, ER stress, oxidative stress, and cell apoptosis were decreased in si*CFB* HK-2 cells under HG conditions. Ceramide emerges as a signal of lipid overload that initiates host cellular stress responses, including ER stress and oxidative stress, ultimately leading to apoptosis induction (35). Therefore, we treated HK-2 cells with C2-Cer and found that improvements in ER stress, oxidative stress, and cell apoptosis mediated by *CFB* knockdown were reversed. This finding illustrated that *CFB* knockdown alleviated PTEC damage via downregulating ceramide under HG conditions. When FB1 was added to *CFB*-overexpressing HK-2 cells, ER stress, oxidative stress, and cell apoptosis were attenuated, suggesting CFB mediates PTEC damage via upregulating ceramide synthases. Moreover, blocking complement activation by *C3* knockdown abolished the effect of CFB on ceramide biosynthesis, suggesting that complement AP activation could cause PTEC damage by affecting sphingolipid metabolism.

Compared with WT diabetic mice, *Cers1*, *Cers2*, and *Cers3* were significantly decreased in *Cfb^–/–^* diabetic mice. Similarly, compared with the control group, *Cers1*, *Cers2*, and *Cers3* were significantly decreased in TCMK-1 cells transfected with si*Cfb* and stimulated with HG. There was a little inconsistency as compared with our cellular experiment in human immortalized cells, in which *CERS1*, *CERS3*, and *CERS6* were reduced in si*CFB* HK-2 cells under the HG environment. This discrepancy may be attributed to the difference in test samples, i.e., renal tubular cells from rodents and humans.

It has been reported that in renal tubular cells, TLR4 could upregulate the expression of CFB and activate the NF-κB signaling pathway (13). The current study found that the NF-κB signaling pathway could mediate inflammation by regulating ceramide biosynthesis. Our results showed that CFB could bind IKBKB, upregulate the expression of NF-κB p65, and promote the translocation of p-p65 from the cytoplasm to the nucleus, resulting in activation of NF-κB signaling. NF-κB is a ubiquitous transcriptional factor regulating innate immune responses (36). The NF-κB signaling pathway activation per se could cause proinflammatory effects, either dependent on or independent of affecting ceramide biosynthesis. A previous study suggested that the LPS/TLR4/NF-κB signaling pathway increases the de novo synthesis of ceramide and upregulates ceramide synthases (37). Our study further extended this finding by demonstrating that *CERS*3 is a direct target gene of NF-κB p65.

There were some limitations in our study. First, renal specimens of HCs were derived from patients with renal carcinoma. Although tissue samples far from the tumor were harvested, the influence of tumor cells and inflammation on the results could not be entirely excluded. However, no obvious abnormality was observed under light, immunofluorescence, or electron microscopy, which minimized such influence. Second, global deletion of *Cfb*, rather than conditional deletion, limited the explanation of CFB function in renal proximal tubular cells.

In conclusion, CFB-mediated tubulointerstitial injury plays an important role in the pathogenesis of DKD. *CFB* deficiency, which blocks the activation of complement AP, alleviates tubular cell damage, including oxidative stress, ER stress, inflammation, and apoptosis, via inhibiting the NF-κB signaling pathway and subsequently blocking the transcription of ceramide synthases, which regulate the biosynthesis of ceramide under HG conditions. The current study provides evidence that CFB may be a promising therapeutic target for DKD.

## Methods

### Human renal biopsies.

A total of 21 patients with renal biopsy–proven DN in the Renal Division, Peking University First Hospital, were enrolled. All patients met the criteria of DN proposed by the Renal Pathology Society in 2010 (2). Patients with other renal diseases, such as IgA nephropathy, membranous nephropathy, and lupus nephritis, were excluded. The general data of these patients are shown in Table 2. We harvested the healthy portions of renal tissues removed from nephrectomy specimens from patients with renal carcinoma as the HCs (*n* = 10). These patients with renal carcinoma did not have diabetes or other kidney diseases, and no obvious abnormality in these specimens was observed under light, immunofluorescence, or electron microscopy. A total of 10 patients with MCD without diabetes were enrolled as the disease control to avoid the confounding effect of proteinuria.

### Animal model.

Eight-week-old sex- and age-matched mice from Nanjing Biomedical Research Institute of Nanjing University were used for this study. *Cfb*-knockout (C57BL/6J background) and WT (C57BL/6J background) male mice were housed in the Animal Center of Peking University First Hospital with a 12-hour light/12-hour dark cycle, pathogen-free conditions, and a bacteria-free diet. The project strategy and identification results of *Cfb^–/–^* mice are presented in Supplemental Figure 2, I–M. We performed agarose gel electrophoresis on DNA isolated from WT and *Cfb^–/–^* mice. *Cfb^–/–^* mice expressed a 650 bp band, and WT mice expressed a 750 bp band. Compared with the WT mice, the mRNA level and protein level of CFB were deficient in *Cfb^–/–^* mice, as demonstrated by RT-qPCR and Western blot. HFD (60% of total calories from fat, D12492; Keao Xieli) was fed to diabetic groups for 4 weeks. Meanwhile, nondiabetic groups were fed a normal chow diet (10% of total calories from fat). After fasting for 4 hours, the diabetic groups received an intraperitoneal injection of STZ (S0130; MilliporeSigma) dissolved in 50 mM sodium citrate buffer (pH 4.5; Solarbio) at 60 mg/kg for 5 consecutive days, while the nondiabetic groups were supplemented with an equivalent amount of 50 mM sodium citrate buffer (38–40). Around 1 week after STZ injection, those abovementioned mice were measured for FPG with a glucose analyzer (Accu-Chek Performa) on tail vein blood samples. Diabetic groups with FPG of over 16.7 mmol/L were included in the study. These mice were grouped as *Cfb^–/–^* diabetic mice (*n* = 6), WT diabetic mice (*n* = 6), *Cfb^–/–^* control mice (*n* = 8), and WT control mice (*n* = 8). All mice were sacrificed and collected for analysis after 21 weeks of STZ injection.

### Anthropometric measurements and laboratory assessment.

Body weight and renal weight measurements of mice were accurate to 0.01 g with electronic scales. Kidney-to-body weight ratio was calculated as left or right kidney weight (g) divided by the body weight (kg). After anesthesia with avertin, heparin-anticoagulated blood of mice was collected from the angular vein. BUN, TCHO, and triglyceride levels in plasma were determined with a UniCel DxC 600 Chemistry Analyzer (Beckman Coulter). 24 hours’ urine was collected in the metabolic cage, and urinary albumin concentration was measured by a mouse albumin ELISA kit (Bethyl Laboratories). Urinary creatinine level was measured by a Creatinine Assay Kit (DICT-500; BioAssay Systems). uACR was calculated as urinary albumin (μg) divided by urinary creatinine (mg).

### Renal histology.

The intensity of C3c staining in patients with DN was semiquantitatively graded on a scale of 0–4 as previously described (10). Tubular atrophy of patients with DN was scored semiquantitatively based on the percentage of the tubulointerstitial compartment that was affected: tubular atrophy (0, no tubular atrophy; 1, <25% lesion; 2, 25%–50% lesion; 3, >50% lesion) and tubulointerstitial inflammation (0, absent; 1, infiltration only in areas related to tubular atrophy; 2, infiltration in areas without tubular atrophy) (2).

Paraffin-embedded mouse tissue sections (4 μm thick) were stained with PAS (BA-4080B; BASO). To quantify glomerular injury and tubulointerstitial injury of each mouse, at least 20 glomeruli or views per section were systematically digitized using a ×400 original magnification and examined by Image-Pro Plus software V.6.0 (Media Cybernetics). Mesangial matrix fraction was expressed as the percentage of mesangial matrix expansion in each glomerulus. Glomerular hypertrophy was measured by analyzing the average percentage of the glomerular area of the observed area. Tubulointerstitial injury of mice was evaluated according to the amount and severity of the loss of brush border, tubular dilation, cast formation, cell lysis, and inflammatory infiltration. Renal tubulointerstitial injury of mice was scored from 0 to 5 as previously described (41): 0, normal; 1, lesion <10%; 2, 10%–20% lesion; 3, 20%–30% lesion; 4, 30%–40% lesion; 5, lesion >40%.

### Immunohistochemistry and immunofluorescence.

Paraffin-embedded kidney tissue sections were immunostained for Bb, CFB, C3a, C3c, C5a, C5aR, and C5b-9. Kidney sections were treated with proteinase K for 10 minutes at 37°C or processed with heat-induced epitope retrieval. Endogenous peroxidases were blocked with 3% hydrogen peroxide for 20 minutes. Kidney sections were incubated in 3% bovine serum albumin (BSA) for 1 hour at room temperature. Primary antibodies against Bb (1:100; Quidel catalog A227), CFB (1:200; Abcam catalog ab192577), C3a (1:150; Abcam catalog ab36385), C3c (1:100; Hycult Biotech catalog HM1078-100UG), C5a (1:300; Abcam catalog ab11878), C5aR (1:2,000; Abcam catalog ab117579), and C5b-9 (1:1,200; Abcam catalog ab55811) were added for overnight incubation at 4°C. Sections were incubated with anti-mouse secondary antibody (ZSBIO catalog PV-9005) and developed using 3,3′-diaminobenzidine (ZSBIO). Slides were viewed under a microscope (Leica) equipped with a digital camera. At least 10 views per section were systematically digitized using a ×400 original magnification and examined by Image-Pro Plus software V.6.0. The IOD or MOD (IOD/total area) was used to represent the deposition of complements in kidneys.

For double-immunofluorescence staining, CFB and specific markers of intrinsic renal cells and inflammatory cells were stained. Frozen sections were incubated with primary antibodies against CFB (1:100; Abcam catalog ab192577), PECAM-1 (1:100; Santa Cruz Biotechnology catalog sc-53411; the marker of glomerular endothelial cells), integrin-α8 (1:100; Santa Cruz Biotechnology catalog sc-365798; the marker of glomerular mesangial cells), synaptopodin (1:100; R&D Systems, Bio-Techne, catalog MAB8977; the marker of podocytes), AQP-1 (1:200; Santa Cruz Biotechnology catalog sc-25287; the marker of PTECs), CD28K (1:100; Santa Cruz Biotechnology catalog sc-365360; the marker of distal tubular epithelial cells), and CD68 (1:100; Abcam catalog ab955; the marker of macrophages). In addition, CFB and C3a were stained for double immunofluorescence. The secondary antibody of Alexa Fluor 488–labeled (AF488-labeled) donkey anti-rabbit IgG (1:200; Jackson ImmunoResearch catalog 711-545-152) and cyanin 3–labeled donkey anti-mouse IgG (1:200; Jackson ImmunoResearch catalog 715-165-150) was added on slices.

HK-2 cells were fixed with 4% paraformaldehyde for 20 minutes and permeabilized with 0.1% Triton X-100 for 15 minutes, then blocked with 3% BSA for 1 hour at room temperature. After incubation with primary antibody of ceramide (1:10; Millipore Sigma catalog C8104) at 4°C overnight, samples were incubated with AF488-labeled goat anti-mouse IgM μ chain (Abcam catalog ab150121) at 37°C for 1 hour. Eventually, the specimens were stained with DAPI. Image-Pro Plus software V.6.0 was used to evaluate the immunofluorescent staining of ceramide. Positive signals were quantified as signal intensity.

### Transmission electron microscopy.

Renal cortical tissues of mice were cut into 3 slices and immediately immersed in 3% glutaraldehyde. Further sample handling was performed by the Laboratory of Electron Microscopy, Peking University First Hospital. Ultrathin sections were obtained from 3 random glomeruli from each mouse, and 20 representative nonoverlapping digital micrographs from each glomerulus were taken under an electron microscope (Hitachi 7700 transmission) at ×10,000 original magnification. The mean FPW and mean width of the cross section of GBM were calculated using Photoshop (Adobe) or ImageJ software (NIH) as previously described (42). In brief, the mean FPW was calculated for each sample, and that value was used to calculate a mean FPW for each group. The arithmetic mean of the FPW was calculated as follows:


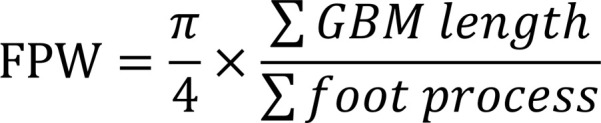


The correction factor of π/4 serves to correct for presumed random variation in the section angle relative to the podocyte’s long axis.

### Tissue RNA extraction and transcriptomics.

Total RNA was extracted using the *mir*Vana miRNA Isolation Kit (Ambion, Thermo Fisher Scientific) following the manufacturer’s protocol. Transcriptome sequencing and analysis were conducted by OE Biotech Co., Ltd. RNA integrity was evaluated using the 2100 Bioanalyzer (Agilent Technologies). The samples with RNA integrity number ≥ 7 were subjected to subsequent analysis. The libraries were constructed using TruSeq Stranded mRNA LTSample Prep Kit (Illumina) according to the manufacturer’s instructions. Then, these libraries were sequenced on the Illumina sequencing platform (HiSeq 2500), and 125 bp/150 bp paired-end reads were generated.

The transcriptome sequencing and analysis were conducted by OE Biotech Co., Ltd. DEGs were identified using the DESeq2 R package functions estimate size factors and nbinomTest. *P* < 0.05 and fold change >2 or < 0.5 were set as the threshold for significantly different expression. Hierarchical cluster analysis of DEGs was performed to explore gene expression patterns. GO enrichment analysis of DEGs was performed using R based on the hypergeometric distribution.

GSEA was performed on transcriptomics, and normalized enrichment score (NES) and FDR were calculated to verify the significant difference for GSEA. GO enrichment analysis for biological processes was performed, and |NES| > 1, *P* < 0.05, and FDR < 0.25 were used as the screening criteria.

### Tissue lipid extraction and quantification.

Tissue lipids were extracted according to modified methods (43). A total of 50 mg tissue sample was homogenized in 500 μL of chloroform/methanol (v/v = 2:1) using a superfine homogenizer. A total of 125 μL of the aqueous solution was added and vortexed for liquid-liquid extraction, followed by repeating 3 times before centrifugation at 1,000*g* for 15 minutes at room temperature. The lower chloroform layer was transferred for secondary extraction and dried under nitrogen.

Liquid chromatography-tandem mass spectrometry method was used for lipid analysis. Data were acquired using a Q Exactive Orbitrap mass spectrometer (Thermo Fisher Scientific) coupled with an UltiMate 3000 (Thermo Fisher Scientific) UHPLC system. LipidSearch software v4.1.16 (Thermo Fisher Scientific) was used to identify and quantify the lipids.

### TUNEL assay.

A colorimetric TUNEL apoptosis assay kit (Beyotime Biotechnology) was used to examine apoptotic cells in paraffin-embedded kidney tissue sections of mice. TUNEL assay was performed using In Situ Cell Death Kit (Roche Diagnostics) to detect apoptotic cells in HK-2 cells according to the manufacturer’s instructions.

### Cell culture.

HK-2 cell line, derived from human immortalized PTECs of kidneys, was purchased from American Type Culture Collection. HK-2 cells were cultured in Dulbecco’s modified Eagle medium/Nutrient Mixture F-12 (DMEM/F12; Gibco, Thermo Fisher Scientific) containing 10% fetal bovine serum (FBS; Gibco, Thermo Fisher Scientific) and 1% penicillin/streptomycin (MilliporeSigma) in a T-25 flask at 37°C in humidified air with 5% CO_2_. The cells were seeded at a density of 1 × 10^5^ cells/well in 6-well plates or 96-well plates. When cell confluence reached 70%, cells were stimulated with HG (final concentration: 30 mmol/L; MilliporeSigma) for 24 hours and then harvested. We set up the osmotic control with mannitol (MilliporeSigma) to avoid hyperosmolarity. To investigate the effect of CFB on HK-2 cells, cells were transfected with si*CFB* (RIBIO) or pCMV-*CFB* (GenScript) using Lipofectamine 3000 (Invitrogen, Thermo Fisher Scientific) dissolved in a non-FBS culture medium. To investigate the role of ceramide synthesis in CFB-mediated PTEC damage, cells were treated with C2-Cer (MilliporeSigma) or FB1 (MilliporeSigma). To investigate the role of complement activation in CFB-mediated ceramide biosynthesis, cells were transfected with LV-*CFB* (GeneChem) for overexpression and si*C3* (RIBIO) for knockdown simultaneously.

The TCMK-1 cell line was purchased from Fuheng Biotechnology (China). TCMK-1 cells were cultured in minimum essential medium (Gibco, Thermo Fisher Scientific). Cells were transfected with si*Cfb* (RIBIO) using Lipofectamine 3000 dissolved in a non-FBS culture medium.

The HEK293T cell line was purchased from Procell Life Science & Technology (China) and cultured in DMEM.

### RNA isolation and RT-qPCR.

Total RNA was extracted from cells or mouse kidney tissues using RNAprep pure Kit (Tiangen Biotech). Primers used for mRNA detection are listed in Table 3. RNA concentration was determined by NanoDrop 1000 (Thermo Fisher Scientific), and 2 μg RNA was reverse-transcribed into cDNA with High-Capacity cDNA Reverse Transcription Kits (Applied Biosystems, Thermo Fisher Scientific). The RT-qPCR analysis was performed with Applied Biosystems 7500 Real-Time PCR System (Thermo Fisher Scientific) using SYBR Green Master Mix (Applied Biosystems, Thermo Fisher Scientific). Relative gene expression was normalized with *β-actin* for humans and *18s* for mice and compared with control groups.

### Western blot.

Samples were incubated for 10 minutes at 95°C in the loading buffer. Samples were then subjected to electrophoresis on 10% SDS-PAGE and transferred to PVDF membranes. The membranes were incubated with primary antibodies of Bb (1:500; Quidel catalog A227), GRP78 (1:1,000; Proteintech catalog 11587-1-AP), CHOP (1:500; Proteintech catalog 15204-1-AP), p-Nrf2 (1:500; Abcam catalog ab76026), cleaved caspase-3 (1:1,000; Cell Signaling Technology catalog 9661), NF-κB p65 (1:1,000; Cell Signaling Technology catalog 8242), p-p65 (1:1,000; Cell Signaling Technology catalog 3033), IκBα (1:1,000; Cell Signaling Technology catalog 4814), p-IκBα (1:1,000; Cell Signaling Technology catalog 9246), IKBKB (1:500; Proteintech catalog 15649-1-AP), histone H3 (1:1,000; Proteintech catalog 17168-1-AP), and β-tubulin (1:2,000; Cell Signaling Technology catalog 2128), followed by horseradish peroxidase–conjugated secondary antibodies (Proteintech catalog SA00001-1 and SA00001-2). Proteins were visualized on autoradiographic film using an ECL Plus Western blot detection system (GE Healthcare, now Cytiva).

### Detection of C3a in the supernatant.

A human complement C3a ELISA kit was used to examine C3a in the supernatant of HK-2 cells according to the manufacturer’s instructions (Qiaoyi Biotechnology).

### Detection of ROS.

We used ROS Assay Kit (Beyotime Biotechnology) to measure the intracellular ROS level. 2′,7′-dichlorofluorescein-diacetate, oxidized to fluorescent dichlorofluorescein by intracellular ROS, was used to quantify the ROS level.

### Detection of SOD.

The content of SOD was determined using the Total Superoxide Dismutase Assay Kit with WST-8 (Beyotime Biotechnology) according to the manufacturer’s instructions.

### Co-IP.

Co-IP was carried out using a Pierce Crosslink Magnetic Co-IP kit (Thermo Fisher Scientific). Briefly, HK-2 cells were lysed and the protein concentration was measured. Then, 500 μg protein in 500 μL supernatant was incubated with 5 μg anti-CFB (Quidel) or anti-IgG (Proteintech) antibodies coated on beads on a rotator overnight at 4°C. The beads were washed to remove unbound material and eluted in a low-pH elution buffer. The precipitate was separated by SDS-PAGE and detected by immunoblotting.

### ChIP assay.

ChIP was performed by SimpleChIP Enzymatic Chromatin IP kit (Cell Signaling Technology). HK-2 cells were cross-linked with 1% formaldehyde for 10 minutes and quenched in 125 mM glycine for 5 minutes at room temperature. After being incubated in cold lysis buffer for 20 minutes, cells were harvested with a cell scraper. DNA was immunoprecipitated from the sonicated cell lysates using an anti–NF-κB p65 antibody (Cell Signaling Technology). The human *CERS3* promoter–specific primers used in PCR are listed in Table 3.

### Promoter activity assay.

The promoter activity of *CERS3* was determined by the Dual-Luciferase Reporter Assay System (Promega). Briefly, pGL3-*CERS3* promoter, pCDNA3.1 (+) or pCDNA3.1 (+)-NF-κB p65 and pRL-SV40 renilla were transfected into HEK293T and HK-2 cells in a 12-well plate using Lipofectamine 3000 for 24 hours. pRL-SV40 renilla vector was cotransfected for normalization of transfection efficiency.

### Data availability statement.

Transcriptomic data produced in this study will be available at NCBI Gene Expression Omnibus database (GSE217153). All data needed to evaluate the conclusions are in the paper and/or the supplemental materials, including the raw data of lipidomics (Supplemental Data 1).

### Statistics.

Data were expressed as means ± standard deviation or median with interquartile range as appropriate. Differences in quantitative parameters among groups were assessed with Student’s 2-tailed *t* test or 1-way ANOVA followed by Bonferroni’s comparison for 2 or more independent samples as appropriate. Correlations among parametric variables were performed using Pearson’s test, while correlations among 1 or more nonparametric variables were assessed using Spearman’s test. A 2-tailed *P* < 0.05 was statistically significant. GraphPad Prism version 7 (GraphPad Software) was used to perform the data analysis.

### Study approval.

Human kidney specimens were obtained from diagnostic renal biopsies performed at the Peking University First Hospital. This study was performed in accordance with the Declaration of Helsinki and was approved by the Research Ethics Committee of Peking University First Hospital. Written informed consent was obtained from each participant. All animal experiments were performed in accordance with guidelines from and with the approval of the Laboratory Animal Ethics Committee of Peking University First Hospital.

## Author contributions

ZJS analyzed statistics and drafted the manuscript. DYC and MHZ designed the study, participated in interpretation of data, and revised the manuscript. MC had full access to all the data and provided final approval of the submitted manuscript. All authors read and approved the manuscript. MC is the guarantor of this work and, as such, had full access to all the data in the study and takes responsibility for the integrity of the data and the accuracy of the data analysis.

## Supplementary Material

Supplemental data

Supplemental data set 1

## Figures and Tables

**Table 3:**
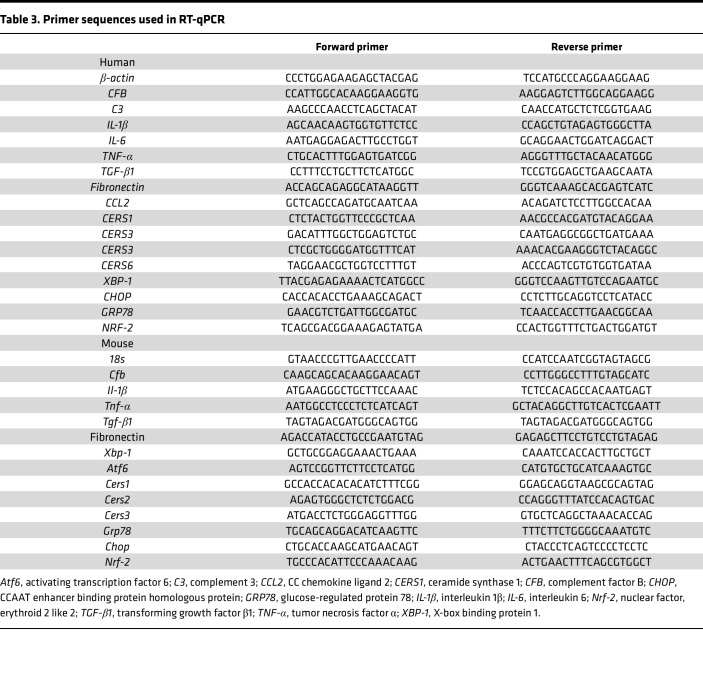
Primer sequences used in RT-qPCR

**Table 1:**
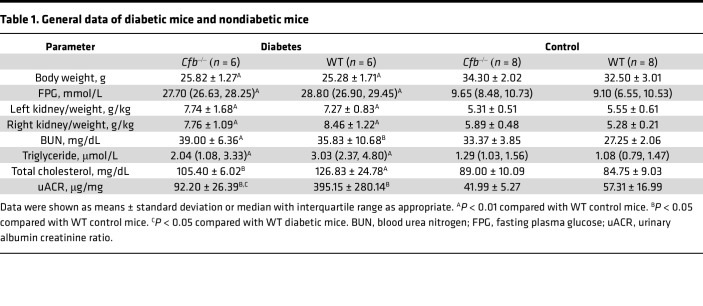
General data of diabetic mice and nondiabetic mice

**Table 2:**
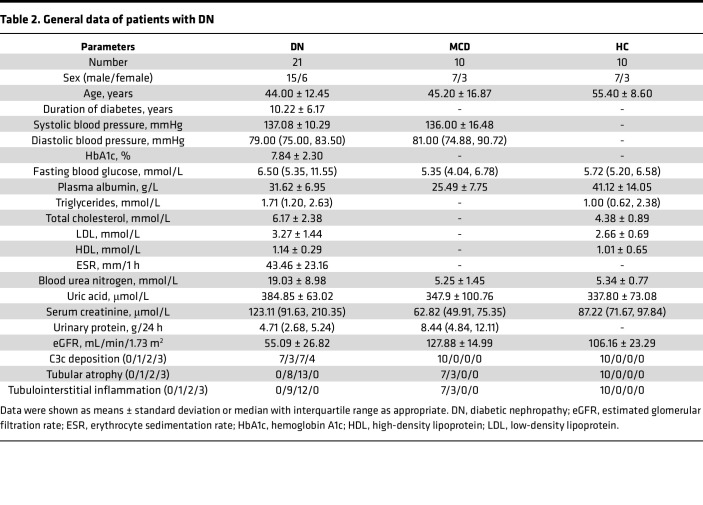
General data of patients with DN

## Version 1 12/22/2022

Electronic publication
