# Supplementary material for: Deficiency of CFB attenuates renal tubulointerstitial damage by inhibiting ceramide synthesis in diabetic kidney disease
Source: JCI Insight. 2022 Dec 22;7(24):e156748. doi: 10.1172/jci.insight.156748 (PMC9869976; doi:10.1172/jci.insight.156748)
Supplement: Supplemental data [file jciinsight-7-156748-s045.pdf]

Supplementary Material

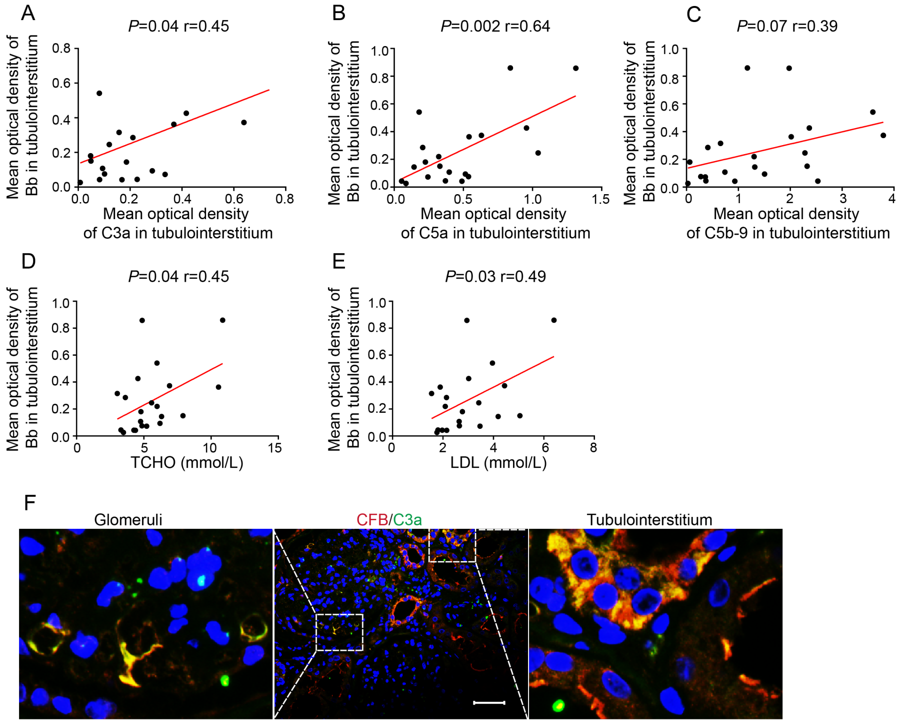

Supplemental Figure 1. Correlation of Bb and other parameters and co-localization of CFB and C3a

Correlation analysis of the MOD of C3a in tubulointerstitium (A), the MOD of C5a in tubulointerstitium (B), the MOD of C5b-9 in tubulointerstitium (C), TCHO (D), and LDL (E) with the level of Bb deposition in the tubulointerstitium of diabetic nephropathy patients. Double immunofluorescence staining of CFB and C3a in the kidney of diabetic nephropathy patient, bar=50μm (F).

LDL, low-density lipoprotein; MOD, mean optical density; TCHO, total cholesterol.

$^{*}P$<0.05; $^{**}P$<0.01; ns (no significance) between groups was determined by Pearson's test or Spearman's test.

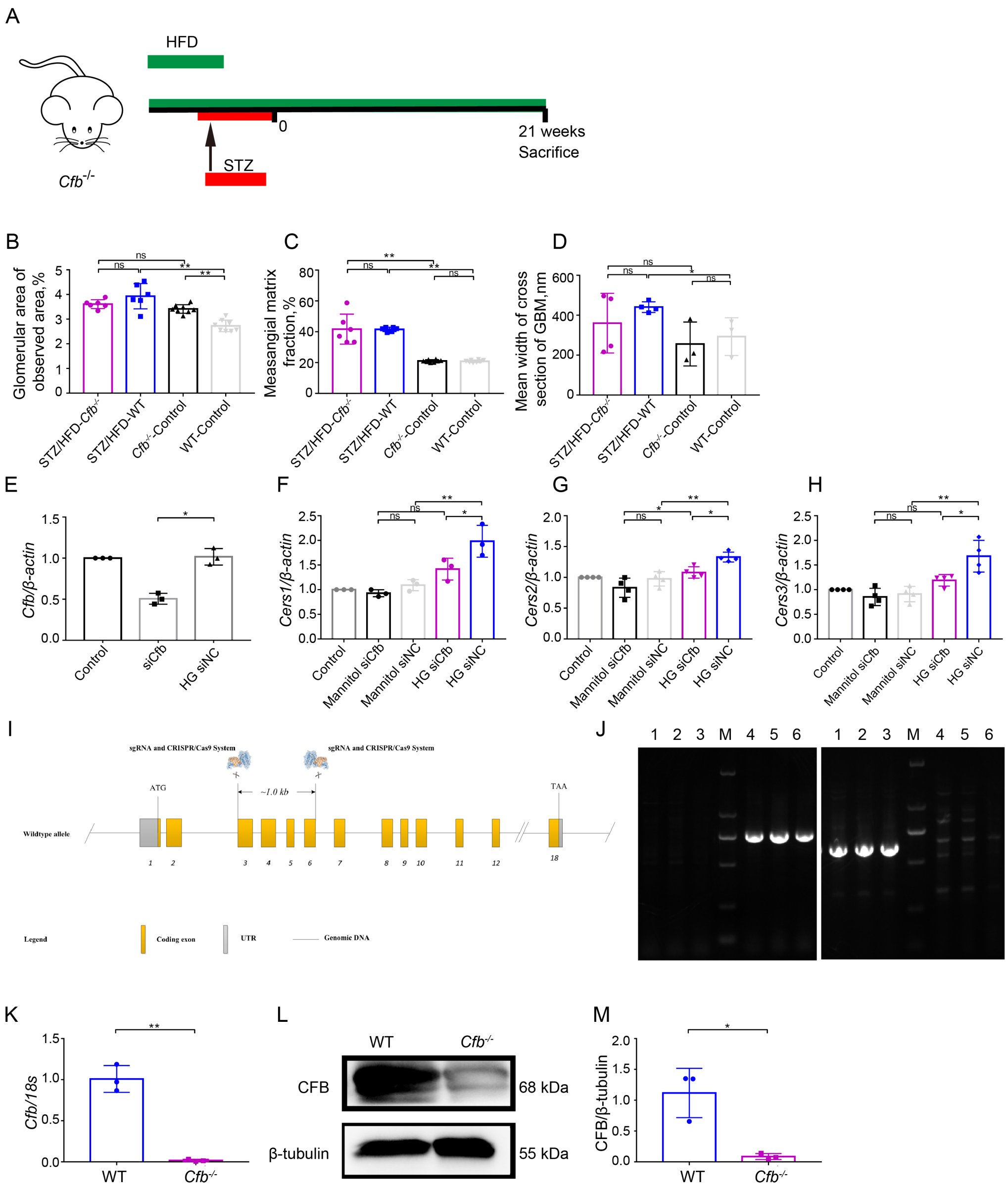

Supplemental Figure 2. Identification of *Cfb* knockout mice

Study design overview. Eight-week-old *Cfb*$^{-/-}$ mice and WT mice were fed a high-fat diet for 1 month followed by an intraperitoneal injection of 60 mg/kg STZ for 5 days. All mice were sacrificed and collected for analysis after 21 weeks of STZ injection (A). Semi-quantitative analysis of glomerular hypertrophy (B, n=6 for diabetic groups, n=8 for non-diabetic groups), mesangial matrix expansion (C, n=6 for diabetic groups, n=8 for non-diabetic groups), and the thickening of GBM (D, n=4 for diabetic group, n=3 for non-diabetic groups) among the above four groups of mice. *CFB* was knocked down by transfecting the siRNA of *Cfb* with lipofectamine 3000 in TCMK-1 cells; RT-qPCR was used to assess the efficiency, n=3/group (E). RT-qPCR was used to measure the expression of *Cers1* (F, n=3/group), *Cers2* (G, n=4/group), and *Cers3* (H, n=4/group) in TCMK-1 cells. The project strategy of *Cfb* knockout mice (I). The agarose gel-electrophoresis of *Cfb* knockout mice (from first to third lines) and WT mice (from fourth to sixth lines, J). RT-qPCR analysis showing *Cfb* mRNA levels in kidneys of *Cfb* knockout mice and WT mice, n=3/group (K). Western blot

demonstrated that CFB expression was barely detected in *Cfb* knockout mice, n=3/group (L), and the ratio of the CFB to β-tubulin was quantified (M).
Cers, ceramide synthases; GBM, glomerular basement membrane; RT-qPCR, quantitative real-time PCR; STZ, streptozotocin; TCMK-1, mouse kidney tubular epithelium cells; WT, wild-type.
$^{*}P<0.05$; $^{**}P<0.01$; ns (no significance) between groups was determined by t-test or one-way ANOVA.

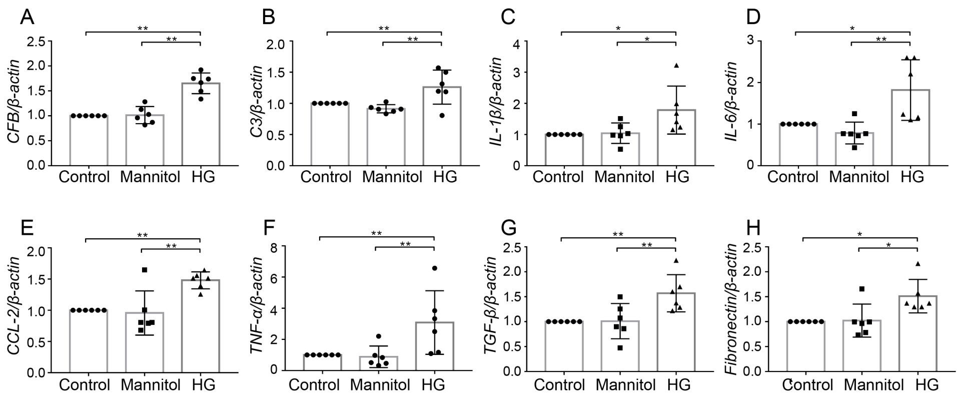

Supplemental Figure 3. Complement components and cytokines were upregulated in HK-2 cells under high glucose conditions

RT-qPCR was used to measure the *CFB* (A), *C3* (B), *IL-1β* (C), *IL-6* (D), *CCL-2* (E), *TNF-α* (F), *TGF-β1* (G), and *fibronectin* (H) mRNA expression in HK-2 cells, n=6/group.

CFB, complement factor B; C3, complement 3; CCL-2, C-C motif chemokine ligand 2; IL-1β, interleukin 1β; RT-qPCR, quantitative real-time PCR; TGF-β1, transforming growth factor beta 1; TNF-α, tumor necrosis factor alpha.

$^{*}P<0.05$; $^{**}P<0.01$; ns (no significance) between groups was determined by one-way ANOVA.

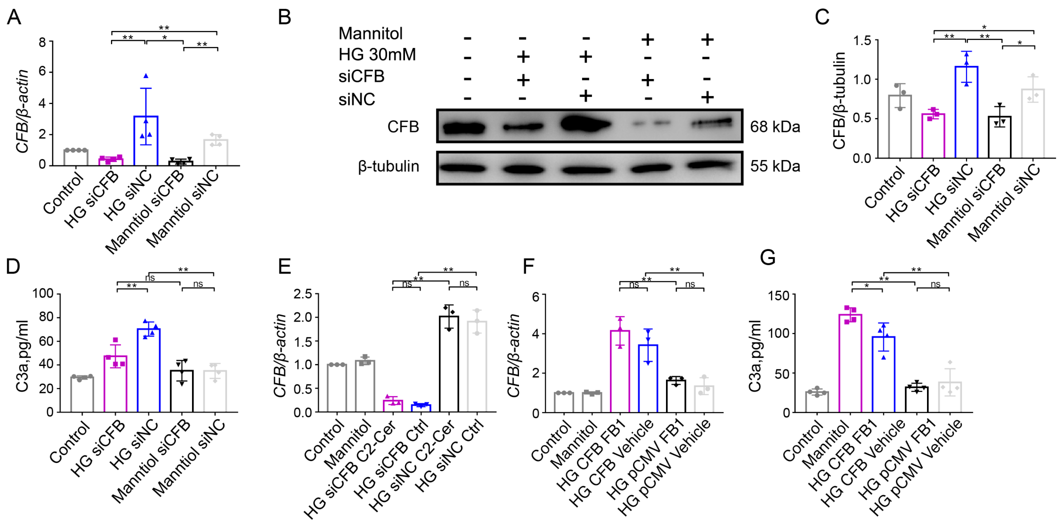

Supplemental Figure 4. The verification of *CFB* knockdown and *CFB* overexpression in HK-2 cells

CFB was knocked down by transfecting the siRNA of *CFB* with lipofectamine 3000. RT-qPCR (A, E; n=4/group) and Western blot (B-C; n=3/group) were used to verify the efficiency. C3a level in the supernatant was detected in HK-2 cells transfected with siRNA of *CFB* (D) and *CFB* (G) plasmid, n=4/group. RT-qPCR was used to illustrate the overexpression of *CFB* in HK-2 cells, n=3/group (F).

RT-qPCR, quantitative real-time PCR.

$^{*}P<0.05$; $^{**}P<0.01$; ns (no significance) between groups was determined by one-way ANOVA.
